# A Bayesian approach to differential edges with probabilistic interactions: applications in association and classification

**DOI:** 10.1093/bioadv/vbad172

**Published:** 2023-11-24

**Authors:** Yu-Jyun Huang, Ying-Ju Lai, Chuhsing Kate Hsiao

**Affiliations:** Division of Cardiology, Department of Medicine, Beth Israel Deaconess Medical Center, Harvard Medical School, Boston, MA 02215, United States; Division of Biostatistics and Data Science, Institute of Epidemiology and Preventive Medicine, College of Public Health, National Taiwan University, Taipei 10055, Taiwan; Division of Biostatistics and Data Science, Institute of Epidemiology and Preventive Medicine, College of Public Health, National Taiwan University, Taipei 10055, Taiwan; Department of Biostatistics, University of Pittsburgh, Pittsburgh, PA 15261, United States; Division of Biostatistics and Data Science, Institute of Epidemiology and Preventive Medicine, College of Public Health, National Taiwan University, Taipei 10055, Taiwan; Institute of Health Data Analytics and Statistics, College of Public Health, National Taiwan University, Taipei 10055, Taiwan; Bioinformatics and Biostatistics Core, Center of Genomic Medicine, National Taiwan University, Taipei 10055, Taiwan

## Abstract

**Motivation:**

Differential network (D-Net) analysis has attracted great attention in systems biology for its ability to identify genetic variations in response to different conditions. Current approaches either estimate the condition-specific networks separately followed by post-procedures to determine the differential edges or estimate the D-Net directly. Both types of analysis overlook the probabilistic inference and can only provide deterministic inference of the edges.

**Results:**

Here, we propose a Bayesian solution and translate the probabilistic estimation in the regression model to an inferential D-Net analysis for genetic association and classification studies. The proposed PRobabilistic Interaction for Differential Edges (PRIDE) focuses on inferring the D-Net with uncertainty so that the existence of the differential edges can be evaluated with probability and even prioritized if comparison among these edges is of interest. The performance of the proposed model is compared with state-of-the-art methods in simulations and is demonstrated in glioblastoma and breast cancer studies. The proposed PRIDE performs comparably to or outperforms most existing tools under deterministic evaluation criteria. Additionally, it offers the unique advantages, including prioritizing the differential edges with probabilities, highlighting the relative importance of hub nodes, and identifying potential sub-networks in a D-Net.

**Availability and implementation:**

All the data analyzed in this research can be downloaded at https://xenabrowser.net/datapages/. The R code for implementing PRIDE is available at https://github.com/YJGene0806/PRIDE_Code.

## 1 Introduction

Complex diseases, such as cancer, are often associated with dysfunctional processes of a group of genes, proteins, or biological pathways. To elucidate the underlying cellular behavior, many studies have focused on the regulated mechanisms of or interactions among molecular events (Peng et al. 2022, Li et al. 2023). This includes the transcriptome research of epistasis, or gene–gene interaction, where the emphasis is on how genes in the same group collaborate to affect human complex traits or react in response to drug interventions (Quan et al. 2018, Wu and Ma 2019). To better understand the difference in cellular function between multiple tissue conditions, differential network (D-Net) analysis has gained much attention in systems biology research (Tu et al. 2021, Leng and Wu 2022). Potential applications of D-Net analysis include identifying the changes in genetic architecture, such as the rewiring relationship under different conditions like disease status or tissue type (Lichtblau et al. 2016, Basha 2020).

Current methods in constructing a D-Net can be roughly classified into two categories. The first adopts a two-stage procedure by estimating separately the network under each condition, followed by post-procedures that compare the difference between two networks to formulate a D-Net. We call this indirectly and separately estimation-based traditional approach, the *IndE*-*based* method. Such analysis is easy to apply since many tools for inferring a single network are available, where the single network is either constructed based on conditional correlations (Meinshausen and Bühlmann 2006, Friedman et al. 2008, Peng et al. 2009) or correlations (McKenzie et al. 2016, Farahbod and Pavlidis 2019). Approaches in the first type utilize conditional correlations and usually assume a multivariate normal distribution, $\text{MVN}(\mu ,\;\Omega =\Sigma^{-1})$, for the gene expression values collected from subjects in the same group, where the group-specific gene regulatory network is determined by the non-zero entries in the precision matrix Ω (the inverse of the covariance matrix Σ); a zero entry corresponds to conditional independence between the paired nodes in the network and therefore implies the absence of a connecting edge within the pair. Specifically, the strength of the edge in this type of Gaussian graphical model for network construction is quantified by the conditional correlation between two genes rather than the pairwise (marginal) correlation. Other studies focused on estimating multiple networks simultaneously by borrowing information from group-specific (condition-specific) networks that are dependent to each other due to shared characteristics, and then derive the differential network. For instance, Danaher et al. (2014) proposed the joint graphical lasso approach in which the lasso and fused lasso penalty induce the similarity of network patterns between groups. Peterson et al. (2015) utilized the Markov random field prior to capture the probability of shared edges between multiple groups. Once the two group-specific networks are derived, then post-procedures are operated on their difference to formulate a D-Net. One common assumption in these IndE-based approaches is that the individual network is sparse. This assumption is useful when exploiting the Lasso penalty in network construction. However, this assumption cannot guarantee that the resulting D-Net is also sparse. In practice, a D-Net is usually sparse since most interacting functions controlling fundamental cellular processes are similar across different tissue types (Neph et al. 2012).

The second type of approach in the indirect estimation category is the correlation-based (Cor-based) methods, in which the D-Net is identified by examining the difference in co-expression pattern between multiple conditions (Farahbod and Pavlidis 2019, Wang and Wang 2022). Precisely, the co-expression pattern is first quantified by estimating the sample correlation within a gene pair under each condition, and the differential correlation network is next constructed by testing if the difference in the Fisher transformations of the group-specific correlation coefficients is zero (McKenzie et al. 2016, Bhuva et al., 2019). This group of methods can measure directly the strength of the rewiring pattern instead of estimating the group-specific networks separately. It should be noted, however, that the D-Net constructed by these correlation-based methods may not be the same as the D-Net defined by the difference in conditional correlation since these two definitions are not identical (Tu et al. 2021). Although the rationale of the differential Cor-based approach is straightforward and easy to implement, these approaches overlook the information of conditional dependency that can be critical when the target is the regulatory pattern of a selected pathway or gene set (Epskamp and Fried 2018, Altenbuchinger et al. 2020).

Methods in the second category of D-Net analysis undertake a direct approach to estimate the D-Net via a direct modeling of the difference in conditional correlations. No learning of the individual group-specific network is required (Zhao et al. 2014, Tian et al. 2016). We call this direct estimation approach the *DE*-*based* method. The DE-based approach assumes edges in the D-Net to be latent variables and estimates them directly via either the constrained $\ell_{1}$ minimization (Cai et al. 2011) or the D-Trace penalized function (Zhang and Zou 2014). The comparative advantages of DE-based over IndE-based methods have been reported (Zhao et al. 2014, Ha et al. 2015, Tian et al. 2016) and can be summarized as follows. First, by combining data from two competing groups in one single analysis, the DE-based methods can utilize a larger sample while the number of parameters is only half that of the IndE-based methods. Such parameter estimation would be more efficient from the statistical point of view. Second, the DE-based approach directly estimates the difference in the conditional dependency between two groups, while retaining the capability to identify differential edges that may be significant in both groups, with the intensity in the same or reverse direction. Note that this cannot be detected with the conditional correlation IndE-based approaches, because this class of methods use the lasso penalty and therefore dichotomize the edges into null or non-null lines in the first stage, and next obtain the D-Net by subtracting the two networks in the second stage. The information on the strength of the conditional dependency is therefore ignored by this class of IndE approaches. Third, the DE-based methods are able to assume directly that the D-Net is sparse, in alignment with most biological information; while the IndE methods, in contrast, may fail to uphold this assumption.

Despite the differences between the IndE- and DE-based methods when conditional correlation is utilized, most of them do not consider the inference as to how strongly or weakly the edge strength affects the response variable and whether the effect is positive or negative. Some carried out the task with feature extraction followed by a regression model or with a mixed graphical model in which the discrete phenotype is included with the continuous gene expression levels in the same network (Lee and Hastie 2015, Sedgewick et al. 2016, Picard et al. 2021). The former does not incorporate the knowledge of network structure and the latter still focuses on the deterministic decision of the edge existence. Investigations regarding the quantification of edge intensity can help the association study of the phenotype or disease as well as the classification study of competing group labels.

This research aims to propose a model to (i) quantify the strength of differential edges in a D-Net, (ii) identify the differential edges by simultaneously considering the rewiring of correlation and conditional correlation patterns, and (iii) provide a probability measure of the existence of differential edges. The proposed Bayesian tool can offer more information than previous methods, extend the Cor-based approaches, and prioritize the importance of identified differential edges with probability. Since the differential network is associated with the “difference” between two competing groups, a logistic regression model comparing two groups with log-odds seems an intuitive choice. Motivated by the application of leveraging quadratic discriminant analysis (QDA) in gene–gene interaction (Xia et al. 2015), here we translate the task of identifying the D-Net into the task of detecting interactions in a logistic regression model and consider the Bayesian logistic regression algorithm to conduct the probabilistic inference. The proposed Bayesian approach contains the Spike-and-Slab Lasso prior (Ročková and George 2018) as the prior distribution of the interaction coefficient parameters, if they pass an a priori screening procedure based on sample correlation coefficients (Huang 2022, Huang et al. 2022), and the resulting posterior probabilities are used to infer the candidate set of edges. To the best of our knowledge, the proposed PRobabilistic Interaction for Differential Edge (PRIDE) is the first study that provides probabilistic inference of differential edges.

## 2 Methods

Let Y be the response variable representing the binary class status k (k=1 or 2) and $X={\left(X_{1},X_{2},\ldots ,X_{P}\right)}^{T}$ be the P dimensional vector denoting gene expression values of P genes. Given Y=k, the X follows a multivariate normal distribution $\text{MVN}(\mu_{k},\Sigma_{k})$, with mean vector $\mu_{k}$ and covariance matrix $\Sigma_{k}.$ For each subject i, i=1,…,N,$\left\{\left(y_{i},x_{i}=\{x_{i1},\ldots ,x_{iP}\}\right)\right\}$ is the corresponding random copy of (Y,X). Then, with simple algebra, the log odds ratio


$$\text{log}(\frac{P(Y=k|X)}{P(Y=\ell |X)})$$

can be expressed as the sum of three terms:


$$\begin{array}{c} \left\{\;\text{log}(\frac{\pi_{k}}{\pi_{\ell }})-\frac{1}{2}\left[\text{log}(\left|\Sigma_{k}\right|)-\text{log}(\left|\Sigma_{\ell }\right|)+\mu_{k}{}^{T}\Sigma_{k}^{-1}\mu_{k}-\mu_{\ell }{}^{T}\Sigma_{\ell }^{-1}\mu_{\ell }\right]\right\}+\\ \left\{X^{T}(\Sigma_{k}^{-1}\mu_{k}-\Sigma_{\ell }^{-1}\mu_{\ell })\right\}+\left\{-\frac{1}{2}X^{T}(\Sigma_{k}^{-1}-\Sigma_{\ell }^{-1})X\right\}. \end{array}$$

Alternatively, this odds ratio can be written as a linear function of X by simultaneously considering all pairwise interaction terms, as in the classical logistic regression model,


(1) $$\text{log}(\frac{P(Y=k|X)}{P(Y=\ell |X)})=\alpha_{0}+\xi^{T}X+X^{T}ΒX.$$

In Equation (1), ξ is the vector of main effects and Β is a P×P symmetric matrix containing the coefficients of the quadratic interaction terms. Comparing the two equations reveals that $\alpha_{0}$ equals the first term,


$$\xi^{T}=\mu_{k}^{T}\Sigma_{k}^{-1}-\mu_{\ell }^{T}\Sigma_{\ell }^{-1},$$

and


$$Β=-(\Sigma_{k}^{-1}-\Sigma_{\ell }^{-1})/2.$$

These equations were the bases in Xia et al. (2015) and Li and Liu (2019), where the test of the D-Net was adopted to replace the test of interaction. We note, however, that the proportionality between Β and $\Sigma_{k}^{-1}-\Sigma_{\ell }^{-1}$ provides crucial information and can reveal the edge existence probability in a D-Net with a Bayesian posterior probability. Such consideration can expand greatly the utility of the above equations.

The rationale of the proposed PRIDE is based on the fact that the magnitude of the interaction coefficient is proportional to the difference of the two precision matrices from the two phenotypic groups, where the difference is commonly defined as the D-Net if multivariate normality is assumed for the node values in the network. In other words, the values of the coefficients can provide information of not only the strength of interaction but also the degree of dependence between two genetic nodes. Another advantage of applying this regression association model for D-Net construction is the ability to utilize various variable selection techniques when the dimension of the D-Net, and hence the number of interaction terms, is ultrahigh. Third, with the Bayesian approach, the probabilistic estimation can be easily implemented to measure the existence uncertainty of the differential edge and even prioritize the edges, highlight the subnetwork, and identify hub nodes.

### 2.1 The Bayesian model and PRIDE

Let (Y,X) be defined the same as above; and without loss of generality, let the gene values be standardized per gene across subjects in the same group, a standard procedure in graphical modeling and D-Net analysis. The formulation of the Bayesian model starts with the binary response $y_{i}∼\mathrm{Ber}(p_{i}),\;i=1,2,\ldots ,N$, with the logit function of the probability $p_{i}$,


$$\;\;\;\;\text{log}(\frac{P(y_{i}=1)}{1-P(y_{i}=1)})=\alpha_{0}+\sum_{1\leq j<k\leq P}\beta_{jk}x_{ij}x_{ik}$$

The complete set of the prior distributions are


$$\begin{array}{l} \;\;\;\alpha_{0}∼N(0,\sigma^{2})\\ \;\;\;\pi (\beta_{jk}|\gamma_{jk})=\gamma_{jk}\times \psi_{1}(\beta_{jk})+(1-\gamma_{jk})\times \psi_{0}(\beta_{jk})\\ \;\;\;\psi_{m}(\beta_{jk})=\frac{\tau_{m}}{2}\text{exp}(-\tau_{m}\left|\beta_{jk}\right|),\;m=0,1\\ \;\;\;\gamma_{jk}∼\mathrm{Ber}(\theta ) \end{array}$$

Here the Bayesian model utilizes the Spike-and-Slab (SSL) prior so that the probabilistic inference can be based on the posterior distributions of $\beta_{jk}$ and $\gamma_{jk}$ to infer the uncertainty of the existence of the differential edges and their relative strength. The SSL prior $\pi (\beta_{jk}|\gamma_{jk})$ contains two components: the slab component is a double exponential distribution


$$\psi_{1}(\beta_{jk})=\frac{\tau_{1}}{2}\text{exp}(-\tau_{1}\left|\beta_{jk}\right|)$$

with a small scale parameter $\tau_{1}$ and the spike component is


$$\psi_{0}(\beta_{jk})=\frac{\tau_{0}}{2}\text{exp}(-\tau_{0}\left|\beta_{jk}\right|)$$

with a large scale parameter $\tau_{0}$. The binary indicator $\gamma_{jk}$ indicates whether the interaction $\beta_{jk}$ comes from the slab or spike, where $\gamma_{jk}=1$ implies that $\beta_{jk}$ is more likely to be generated from a distribution for a strong effect (i.e. $\beta_{jk}\neq 0$) than the null effect ($\beta_{jk}\approx 0$). Therefore, a large posterior probability of $\gamma_{jk}=1$ would be supporting evidence of the existence of a differential edge. This probability is a measure of the uncertainty of the existence. Previous research has addressed the test of $\beta_{jk}=0$ to determine if the edge between node j and k is different for the two condition groups. No focus has been placed on the estimation perspective.

### 2.2 Screening strategy and algorithm

The number of all possible interactions is φ=P(P−1)/2, which is of the order $O(P^{2})$. However, there is no need to estimate every interaction parameter since the D-Net is usually sparse. Therefore, the following two-stage screening procedure is adopted to reduce the number to a more reasonable and computationally affordable value. In the first stage, a set of S candidate interactions are screened by the sample Pearson correlation (Fan and Lv 2008). This step incorporates the strength of the differences in sample correlation between two groups into PRIDE. In addition, since the difference in the Fisher-transformed sample correlation is tested as interaction in logistic regression (Bien et al. 2015), it offers a promising way to reduce the computational burden in estimating a large number of non-differential interactions. The interactions in this set are next fitted with the Bayesian logistic regression model. The algorithm is summarized below.

**PRIDE**

**First stage: screening**

1. Compute the pair-wise sample Pearson correlation per response group:

${\hat{\rho }}_{jk}^{1},\;i\leq j<k\leq P$ for Y=1 and ${\hat{\rho }}_{jk}^{0},\;i\leq j<k\leq P$ for Y=0.
2. Compute the difference in sample correlation between two groups:

$\Delta =\left\{\Delta_{jk}:\;\Delta_{jk}={\hat{\rho }}_{jk}^{1}-{\hat{\rho }}_{jk}^{0},\;1\leq j<k\leq P\right\}.$

3. Order the absolute values of all $\Delta_{jk}$ from greatest to least and retain the top S differences in the candidate set SR,

$SR=\left\{(j,k):\;{\left|\Delta_{jk}\right|}^{\left(1\right)},{\left|\Delta_{jk}\right|}^{\left(2\right)},\ldots ,{\left|\Delta_{jk}\right|}^{\left(S\right)}\right\},$

where the superscript (i), i=1,…,S, denotes the order.

**Second stage: estimation with the Bayesian model**

1. The logit function can now be rewritten as
  $$\text{log}(\frac{P(y_{i}=1)}{1-P(y_{i}=1)})=\alpha_{0}+\sum_{(j,k)\in SR}\beta_{jk}x_{ij}x_{ik}$$
2. Compute the posterior distributions of the above $\beta_{jk}$ and $\gamma_{jk}$ for inference.

**Figure 1.**
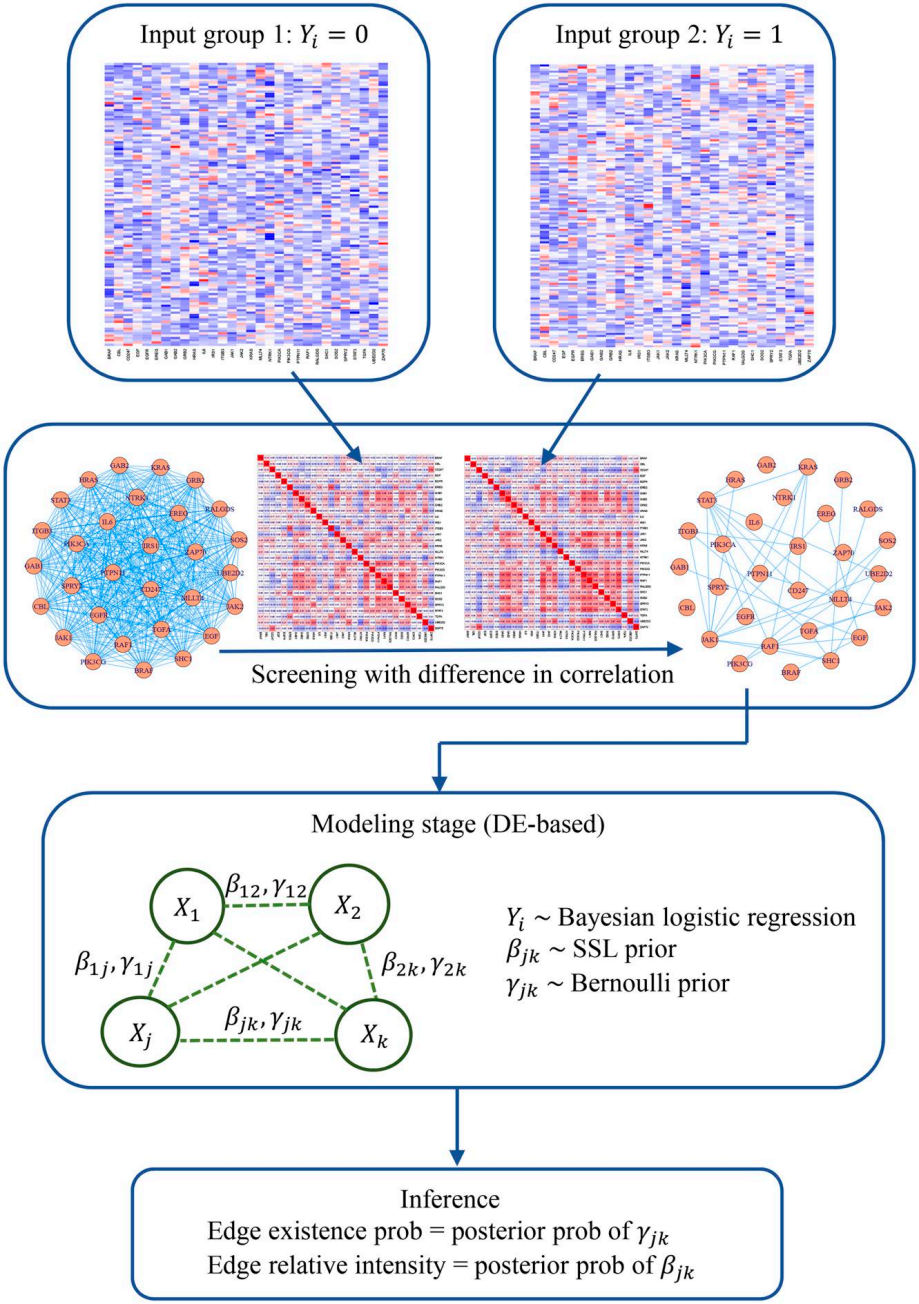
The diagram of PRIDE. The PRIDE algorithm starts with the input of two gene expression data matrices, then a pre-defined structure of the D-Net was constructed based on a screening procedure, followed by a Bayesian logistic regression model and posterior inference on the edge existence probability and relative intensity.

Two strategies are available to select the number S. The first one is related to the sample size N, where S can be taken as the integer closest to the ratio of the sample size to the number of nodes N/P. The same choice has been considered in Fan and Lv (2008) and Hung et al. (2016) in ultrahigh dimensional variable selection problems. The second strategy relates to the sparsity of a single network, which usually ranges between 5% and 10% based on estimates with networks in public databases (Leclerc 2008, Huang et al. 2022). Therefore, it is reasonable to assume the sparsity of D-Net is not larger than these values. That is, one can use either S=5%×P(P−1)/2 or10%×P(P−1)/2. The hyperparameters in the spike-and-slab prior distributions are set as $\tau_{0}=20$ (spike) and $\tau_{1}=2$ (slab), respectively, to reflect the vague information about $\beta_{jk}$. The choice of these parameter values has little effect in the probabilistic inference, as demonstrated in Huang et al. (2022). The prior probability θ of the existence of a differential edge is set at 0.7 because it has passed the screening procedure. All computations are carried out with Markov chain Monte Carlo (MCMC) algorithm implemented with the R package *R2OpenBUGS* to generate posterior samples for inferences. The resulting posterior probability of $\gamma_{jk}=1$ will denote the uncertainty of the existence of an edge in the D-Net. A threshold, say 0.5, is adopted to imply existence. The diagram of the PRIDE is displayed in Fig. 1.

## 3 Results

### 3.1 Simulation studies

The performance of PRIDE is compared with existing tools, including the Cor-, DE-, and IndE-based methods in this section. Three scenarios are considered in the simulation studies. The first two (M1 and M2) are for the case where the D-Net is sparse, with an even lower sparsity than that of the individual group-specific networks. The scenario M1 contains a D-Net resulting from differences in the structure of the individual group-specific networks and the D-Net in M2 results from differences in intensity of the corresponding edge in each group. The third scenario M3 is designed for the D-Net whose sparsity is not much lower than that of the individual networks, and to investigate the impact of network size.

For the Cor-based approaches, we considered the DGCA (McKenzie et al. 2016) and the EBcoexpress (Dawson and Kendziorski 2012) in the simulation studies. Two types of *P*-value adjustment procedures, the Benjamini–Hochberg (BH) and Bonferroni correction (Bonf) methods, are utilized in DGCA to detect the differential correlation edges (*DGCA* R package). The hard posterior probability threshold for EBcoexpress (EB) to identify the differential correlation edges is set as 0.9, which is the default setting in the R package *dcanr* used in the analyses.

Of the DE-based methods, D-Trace, logistic Lasso, and logistic Lasso with screening (S-Lasso) are considered. D-Trace is carried out with the R package *DiffGraph*, where the tuning parameter is determined by searching on a 0.05 grid scale between 0.1 and 0.45 and selecting the one with the largest F1-score. This strategy may lead to overfitting so the superior performance of D-Trace can be expected. The logistic Lasso is implemented using the R package *glmnet*, and the tuning parameter is selected by 10-fold cross-validation. The screening strategy for S-Lasso is identical to that for PRIDE for a fair comparison. Throughout the simulation studies, the posterior probability threshold 0.5 is used to determine the existence of a differential edge in the PRIDE framework.

For IndE-based tools, the estimation of each separate network is required and therefore we consider the graphical Lasso (Friedman et al. 2008), neighborhood selection (Meinshausen and Bühlmann 2006), and Space (Peng et al. 2009) approaches. For these approaches, we use, respectively, glasso and the default selection for the argument Method in the R package *huge* with the tuning parameter chosen based on the rotation information criterion (ric); and the R package *space* with the tuning parameter set by default.

#### 3.1.1 Simulation settings of M1 and M2

**Figure 2.**
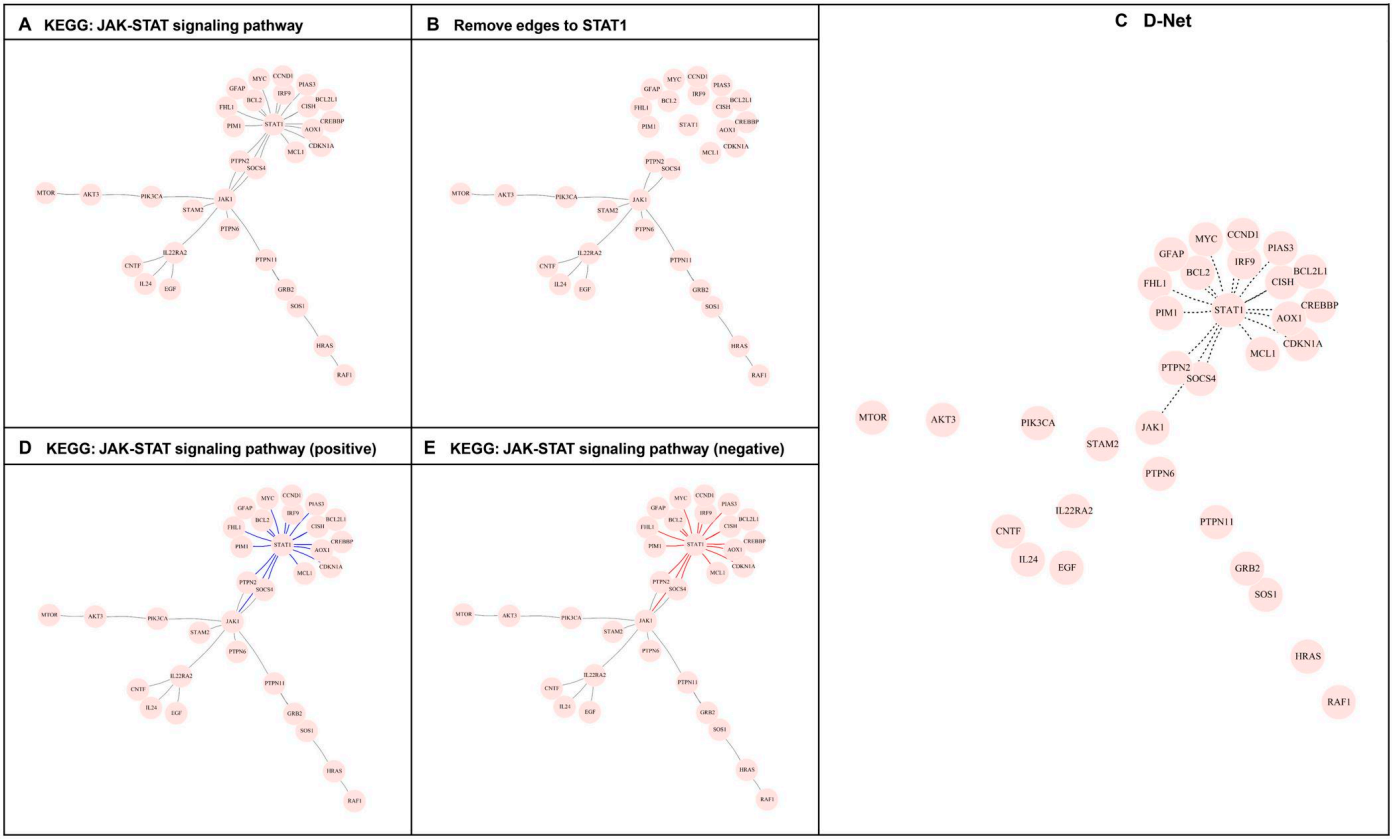
The schematic overview of the D-Net for simulation M1.1. and M2.1. **A**: The original JAK-STAT signaling pathway. **B**: The pathway with the edges connecting to STAT1 removed. **C**: The resulting D-Net. **D**: The JAK-STAT signaling pathway with edges of positive (colored blue) intensity. E: The JAK-STAT signaling pathway but with edges of negative (colored red) intensity.

Under each scenario, the structures of the JAK-STAT and MAPK signaling pathways from the KEGG pathway database as well as structure of the EGFR pathway from the protein–protein interaction (PPI) network database are considered. For instance, Fig. 2C represents the D-Net from two networks, one with and one without the STAT1 gene node in the JAK-STAT pathway (Fig. 2A versus Fig. 2B) as an example of the scenario M1. Fig. 2C also represents the D-Net from two networks with edges of different levels of intensity (Fig. 2D versus Fig. 2E), an illustration of the scenario M2. In all cases, the regulatory structures of the two KEGG pathways were obtained by implementing the algorithm in Chang et al. (2020), and the EGFR network containing edges with a combined score larger than 0.99 was downloaded from the STRING database.

Tables 1 and 2 list the details of the four settings under each scenario (M1.1–M1.4 and M2.1–M2.4), including the database (KEGG or PPI) from which the pathway was obtained, name of the original network (JAK-STAT, EGFR, or MAPK), the number of nodes (32, 51, or 115), the difference in the two competing networks (structure or intensity), number of differential edges, sparsity of the D-Net, and the true intensity of differential edge. Note that the sparsity of the assigned D-net is low, lower than the sparsity of the group-specific network (details in Supplementary Tables S1 and S2). The number of interactions passing the screening procedure before the implementation of the Bayesian model and other details are listed in Supplementary Table S3. Under each setting, the sample size is 250 per group and the number of replications is 100 for the evaluation of performance. In the simulation, once the network structure is fixed, the adjacency matrix is computed and the R package *huge* is employed to create the precision matrix and generate sample values from the corresponding multivariate normal distribution. The number of screened interactions S is listed in Supplementary Table S3.

**Table 1.** Four differential D-Nets under scenario M1, including M1.1, M1.2, M1.3, and M1.4 from top to bottom.

Database	Name	*P*	Node	#	s (%)	diff
KEGG	Jak-Stat	32	STAT1	17	3.4	0.219
STRING	EGFR	51	EGFR	16	1.3	0.206
KEGG	MAPK	115	MAPK14	15	0.2	0.196
KEGG	MAPK	115	MAPK14	15	0.5	0.196
			MAPK8	15		

The information includes the database where the network is retrieved, the name of the pathway or PPI, the number of nodes in the network (P), the node connecting to the differential edge (Node), the number of differential edges (#), the sparsity of the D-Net (s), and the true intensity of the differential edges (diff).

**Table 2.** Four differential D-Nets under scenario M2, including M2.1, M2.2, M2.3, and M2.4 from top to bottom.

Database	name	*P*	Node	#	s (%)	diff
KEGG	Jak-Stat	32	STAT	17	3.4	0.22
STRING	EGFR	51	EGFR	16	1.3	0.22
KEGG	MAPK	115	MAPK14	15	0.2	0.22
KEGG	MAPK	115	MAPK14	15	0.5	0.22
			MAPK8	15		

The information includes the database where the network is retrieved, the name of the pathway or PPI, the number of nodes in the network (P), the node connecting to the differential edge (Node), the number of differential edges (#), and the sparsity of the D-Net (s). Note that the true intensity of the differential edges is 0.11−(−0.11)=0.22.

#### 3.1.2 Performance comparison under M1 and M2

Various measures are considered to evaluate the performance, including the number of true positives (TP) defined as the number of true differential edges that are correctly identified, false positives (FP) as the number of true non-differential edges incorrectly identified, and false negatives (FN) as the number of true differential edges not identified, as well as the sensitivity (SEN), specificity (SPE), false discovery rate (FDR), Matthew correlation coefficient (MCC), and F1-score (F1), where F1 is defined as


$$F1=\frac{2\times \text{TP}}{2\times \text{TP}+\text{FP}+\text{FN}}.$$

**Figure 3.**
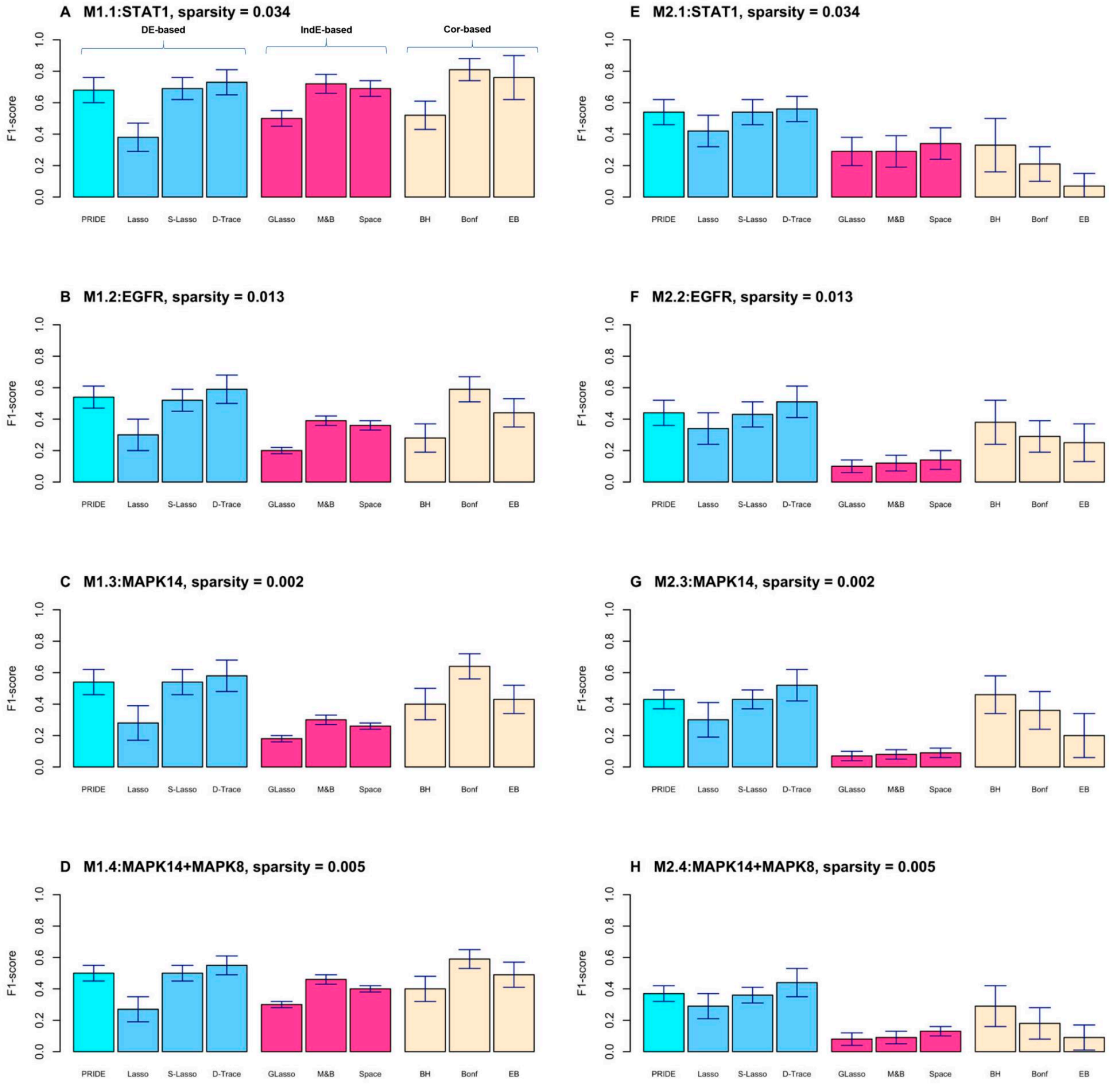
F1-score (Y-axis) under scenario M1 and M2. The results of scenario M1 and M2 are shown in the left (**A–D**) and right (**E–H**) panel, respectively. The Y-axis denotes the average F1-score across 100 replications, with the error bar as the standard error. The DE-based methods are denoted in blue (PRIDE in light blue), the IndE-based methods are in pink, and the correlation-based (Cor-based) are in yellow.

These performance measures are reported in Fig. 3.

The four subfigures in the left panel of Fig. 3 are the four settings in scenario M1. In general, all tools perform better when the sparsity is larger (0.034 in Fig. 3A) but worse if lower (0.013, 0.002, and 0.005 in Fig. 3B–D, respectively). However, the DE-based methods (colored blue) are more robust against a decrease in sparsity, followed by the Cor-based methods (colored yellow). The IndE-based methods (colored pink), though assuming low sparsity for each individual group-specific network, cannot control the sparsity of the D-Net, and therefore produce a large FP in the D-Net (Supplementary Table S4). Interestingly, due to the large differences in sample correlation for those truly differential edges (around 0.4–0.6) in this setting, the Cor-based approaches (colored yellow) show a comparable performance with the DE-based methods. This concordance can be found in later real data analysis as well.

Among the group of DE-based methods in Fig. 3, D-Trace performs best in terms of all measures except sensitivity (Supplementary Table S4). However, PRIDE is comparable to D-Trace, which could be overfitting, and outperforms D-Trace in sensitivity. Furthermore, PRIDE provides existence probabilities and makes possible inference of the intensity with posterior distributions, delivering more information than the other tools. In addition, the comparison between logistic Lasso (denoted Lasso in the figure) and logistic Lasso with screening (denoted S-Lasso in the figure) reveals the advantage of the screening procedure in reducing the computational burden and the control of false positives (Supplementary Table S4). Comparison based on other measures is summarized in Supplementary Table S4.

The results under scenario M2 are presented in the right panel of Fig. 3. Again, it is obvious that the DE-based methods performed much better than the IndE-based methods, with the Cor-based methods ranked between these two. The IndE-based tools could not handle the case when the effects in two competing groups are of different directions. Among the DE-based methods in the left group, the D-Trace provided the largest F1-score and smallest FP (Supplementary Table S5) but often the lowest sensitivity. In contrast, the performance of PRIDE is more stable across various measures.

When comparing the results in simulation M1 versus M2 (left versus right panel in Fig. 3), it is noted that both the IndE- and Cor-based approaches have a noticeable decrease in F1-score in M2. This is because the differences in sample correlation and sample conditional correlation between two groups are only between 0.2 and 0.3 for those truly differential edges. The magnitude is nearly half of that in M1. This smaller gap could cause the loss of power. In contrast, PRIDE performs better in terms of F1-score in M1 and M2 and is more robust to the reduced difference.

In summary, the DE-based methods can guard against the case when the sparsity in D-Net is smaller than that in the individual network and can deal with the case when the intensity levels in two group-specific networks are not similar.

#### 3.1.3 Comparison under scenario M3

Under M3, the D-Net between an AR(1) and AR(2) network is to be identified. The number of nodes is set at 25, 50, or 70. The partial correlation is set at 0.3 for the first-order neighbors in both networks, and the second-order neighbor in the AR(2) structure is set at 0.22. The D-Net, therefore, contains all the second-order interactions in the AR(2) structure. The corresponding sparsity and other information are in Supplementary Table S6. The pattern of performance (Supplementary Fig. S1 and Supplementary Table S7) is the same as that in M1 and M2. That is, the DE-based tools perform better than the IndE-based and Cor-based methods, and among the DE methods, D-Trace is the best but PRIDE is comparable or better if considering F1-score, FP and sensitivity as the evaluation criteria. Additionally, the performance of all methods is impacted by the increase in P, however, the DE-based methods are more robust to the increase in network size.

### 3.2 Data applications: the cancer genome atlas glioblastoma study

The glioblastoma (GBM) is a highly malignant and lethal brain tumor with poor prognosis and short survival time. The etiology is still unclear and its high heterogeneity may be related to differential responses to treatments (Park et al. 2019 in Supplementary notes T1). Several studies have focused on rewiring the molecular interaction mechanism in different tumor subtypes for precision medicine. Here the proposed PRIDE is applied to the GBM data from TCGA (The Cancer Genome Atlas Research Network 2008 in Supplementary notes T1) for the D-Net analysis, where the data were downloaded from https://xenabrowser.net/datapages/. The gene expression values were generated from the Affymetrix HT Human Genome U133a microarray platform with log2(RMA) mRNA values. To reduce the influence of heterogeneity from the population structure, only those patients of European ancestry with the Mesenchymal (136 subjects) or Proneural (123 subjects) subtype were retrieved. These two subtypes have been reported to be in two separate clusters (Steponaitis and Tamasauskas 2021 in Supplementary notes T1) and share different prognoses (Sidaway 2017; Teo et al. 2019 in Supplementary notes T1). A set of 30 genes showing association with EGFR was selected from the STRING database (Version 11.0 b; https://string-db.org/) to construct the differential network. The screening procedure selected 40 interactions to control the sparsity at 0.1.

**Figure 4.**
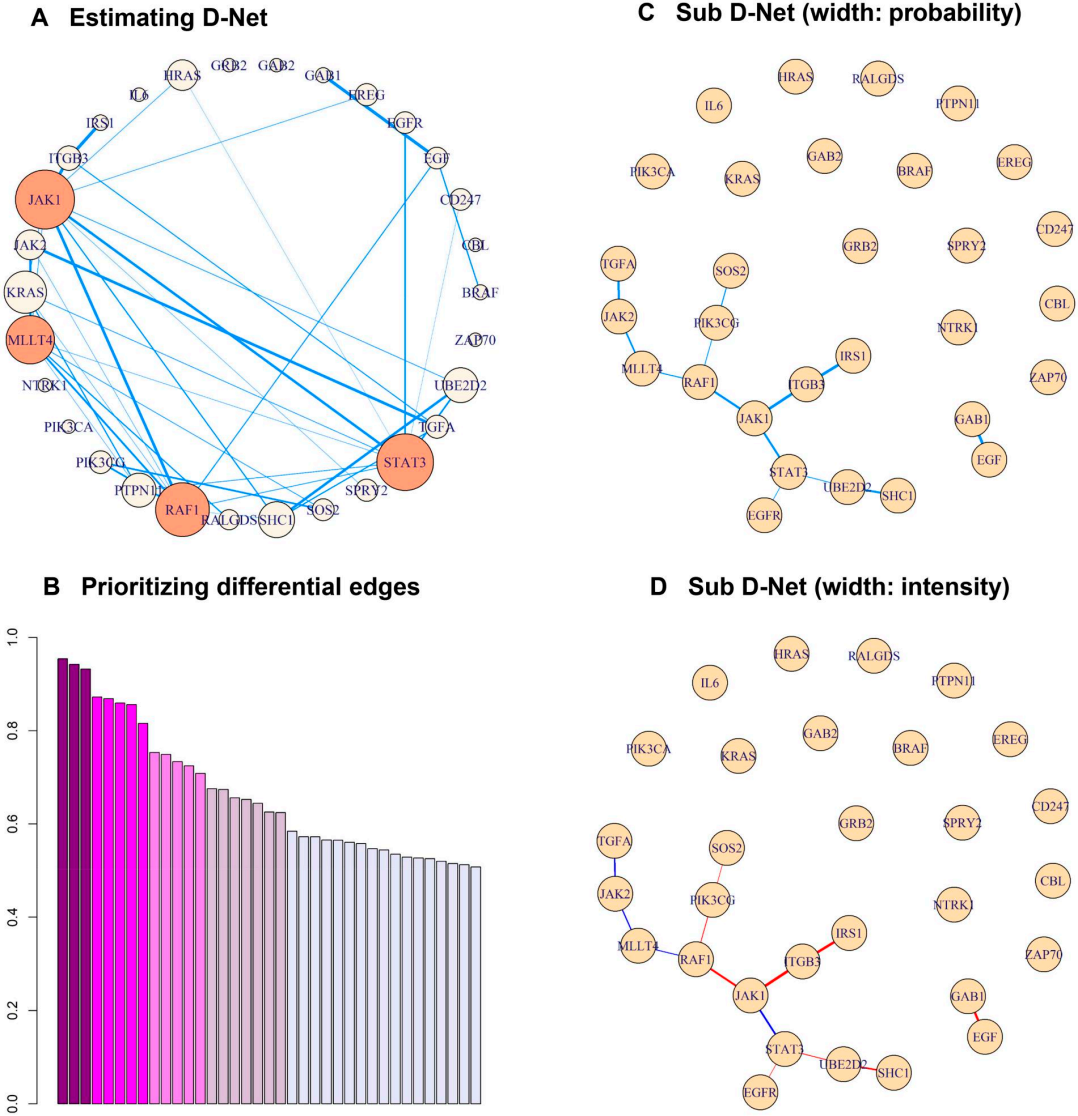
D-Net analysis of two TCGA GBM subtypes. **A**: The D-Net contains edges with posterior probability larger than 0.5. The width corresponds to the estimated probability. The node (gene) size is proportional to the number of differential edges connected to it. Four hub nodes are highlighted. **B**: The ordered posterior probability of the 37 differential edges, with different colors for different thresholds 0.9, 0.8, 0.7, 0.6, and 0.5. **C**: A subnet of **A** with only 13 differential edges, each with probability larger than 0.7. The width corresponds to the probability. **D**: The same subnet in **C** but the width corresponds to the posterior mean of $\beta_{jk}$. A red edge indicates higher intensity in the Proneural than in the Mesenchymal subtype and a blue edge indicates the reverse.

The D-Net identified by PRIDE is displayed in Fig. 4, where Fig. 4A contains 37 differential edges with probability larger than 0.5 and Fig. 4C contains 13 edges with probability larger than 0.7. With different thresholds, one can identify subnets in the D-Net as demonstrated here. Noted that the four hubs (JAK1, STAT3, RAF1, and MLLT4) identified in Fig. 4A also appear in the center of the subnet in Fig. 4C with connecting edges of wide width (large existence probability) and large average intensity level (Fig. 4D). The ordered posterior probabilities are displayed in Fig. 4B, which can be considered as prioritized targets for future drug development. Other methods are applied to the GBM study, and Table 3 lists the results when compared with the 13 edges identified by PRIDE in Fig. 4C. Note that the findings are fairly consistent within the DE-based methods, a pattern observed in the simulation studies as well.

**Table 3.** The identified 13 differential edges with probability larger than 0.7 under nine different methods.

G1	G2	prob	Mean	S-Lasso	Lasso	D-Trace	GLasso	M&B	Space	BH	Bonf	EB
EGF	GAB1	0.954	0.527	1	1	1	0	0	0	0	0	0
IRS1	ITGB3	0.942	0.567	1	0	1	0	0	0	0	0	0
ITGB3	JAK1	0.932	0.626	1	0	1	1	1	1	1	0	0
JAK1	RAF1	0.872	0.464	1	1	1	1	1	1	1	1	1
JAK2	TGFA	0.868	−0.367	1	0	1	1	1	1	0	0	0
SHC1	UBE2D2	0.859	0.395	1	1	1	1	0	0	1	0	1
**JAK1**	**STAT3**	**0.856**	**−0.439**	**0**	**0**	**0**	**0**	**0**	**0**	**1**	**1**	**0**
JAK2	MLLT4	0.816	−0.348	1	1	1	0	0	0	0	0	0
MLLT4	RAF1	0.753	−0.283	1	1	1	0	0	0	1	0	1
PIK3CG	SOS2	0.749	0.219	1	1	1	1	1	1	0	0	1
PIK3CG	RAF1	0.733	0.244	1	1	0	0	0	0	0	0	1
STAT3	UBE2D2	0.724	0.247	1	1	1	1	0	0	1	1	1
EGFR	STAT3	0.708	0.217	1	1	1	0	0	0	0	0	1

The first two columns (G1 and G2) are the gene nodes connected by the differential edges. The third column (prob) indicates the posterior probability $P(\gamma_{jk}=1|.)$and the fourth column (mean) lists the posterior mean of $\beta_{jk}$. A “1” in the last six columns indicates that the edge is also identified by the corresponding approach and a “0” indicates it is not. Bold values emphasize the gene pair identified only by BH and Bonf methods.

**Figure 5.**
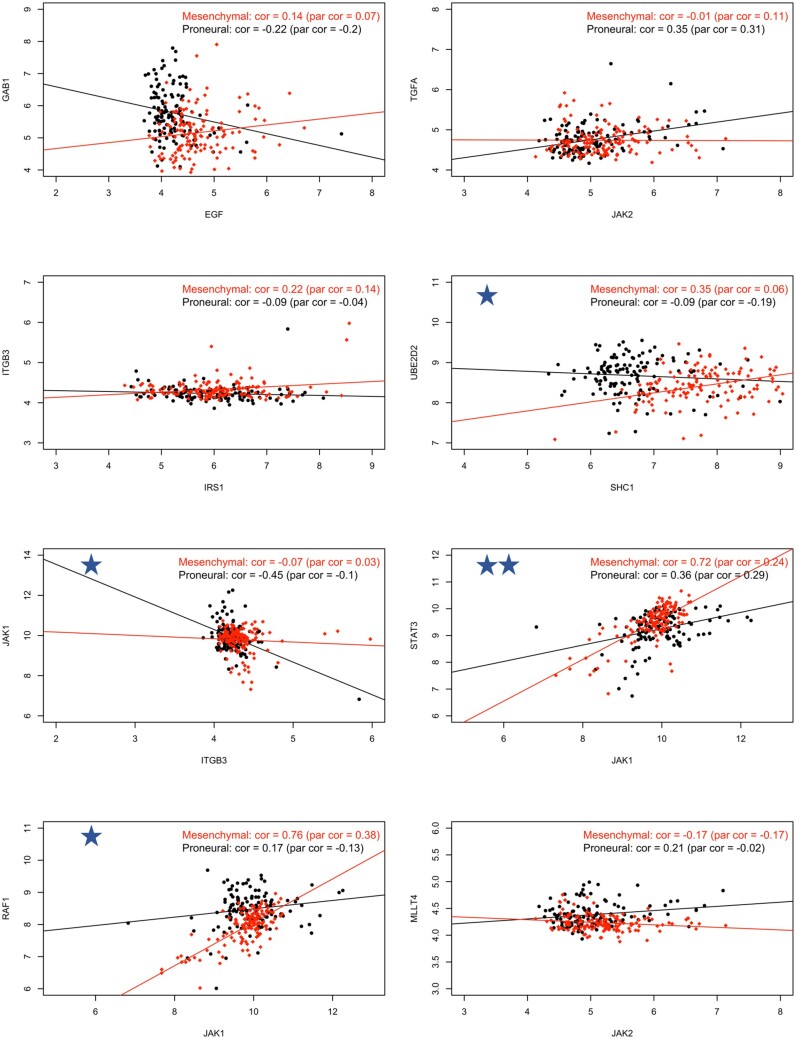
Scatter plots of co-expression patterns of the top 8 gene pairs identified by PRIDE. The sample correlation (cor) and partial correlation (par cor) between the paired genes are calculated per each subtype. Red dots are subjects of the Mesenchymal subtype, and black dots indicate the Proneural subtype. The star symbol on the top-left corner in four subfigures implies that this pair is also identified as a differential correlation edge by the DGCA method. The two-star symbol indicates that the gene pair JAK1-STAT3 is identified only by PRIDE and DGCA.

The co-expression pattern of the top eight pairs of genes with the highest posterior probability calculated by PRIDE is summarized in Fig. 5. Among them, four pairs of genes are highlighted with stars. These four are identified as differential correlation edges by the DGCA method. Since these four pairs all show large difference in sample correlations across groups (ranging between 0.4 and 0.6), the two methods provide consistent findings, a pattern already observed in the simulation studies M1. However, for the top two gene pairs identified by PRIDE, the EGF-GAB1 and IRS1-ITGB3, these two pairs were not detected by the differential correlation methods. This could result from the small difference in sample correlation.

One differential edge worth discussing is the one between JAK1 and STAT3, which is identified only by PRIDE and the Cor-based methods. The group-specific correlation of this pair differs significantly between two competing phenotypic groups (0.72 versus 0.36), where each shows a strong partial correlation coefficient (0.24 versus 0.29). The DGCA Cor-based method tests the difference in the strength of the correlation and detects this differential edge. In contrast, other D-Net analyses focus on whether the edge exists in each group-specific network and not the difference in intensity; therefore, these other analyses indicate null differential edge within the pair. This demonstrates again the importance of considering the magnitude of differential edges in D-Net analysis. In addition, the interaction between JAK1 and STAT3 has been documented in literature and has been the functional target of drug development. Examples include AZD1480 to inhibit the growth of solid tumors such as GBM (Qureshy et al. 2020, Ou et al. 2021 in Supplementary notes T1). More comparison such as a Venn diagram of the intersection of the findings among the methods is shown in Supplementary Figs S2–S4.

The four hub genes identified by PRIDE have been reported to associate with GBM progression. For example, the oncogenic activity and immunosuppression of STAT3 can regulate glioma stem cells and may correspond to the mediation of chemoresistance (Kim et al. 2014, Ou et al. 2021 in Supplementary notes T1). The identified differential interaction between STAT and EGFR is supported by the increasing evidence that STAT signaling may be dysregulated by the amplification of EGFR (Qureshy et al. 2020, Ou et al. 2021 in Supplementary notes T1). We also identified the differential interaction between JAK1 and STAT3 with PRIDE, where evidence in the literature reports that JAK inhibitors can decrease the activation of STATs, and their interaction has been demonstrated as a viable target for drug development, such as AZD1480 for inhibiting the growth of GBM tumors. Other research has shown that the oncogene RAF1, as targeted by microRNA miR-7-5p, is associated with microvascular proliferation of GBM (Liu et al. 2014 in Supplementary notes T1), and the finding that miR-424 actives RAF1 in ERBB signaling which may be associated with apoptosis in GBM cells, indicating a possible target on RAF1 for antitumor drug research (Gheidari et al. 2021 in Supplementary notes T1). Moreover, the MLLT4 gene not only has been illustrated to participate in the RAS signaling pathway as the factor of cell junctions related to progression of GMB but also been demonstrated as a crucial predictor to efficiently classify prognostic categories of GBM patients (Yang et al. 2019 in Supplementary notes T1). Previous studies that supported the biological insight found in this real data analysis are listed in Supplementary note T1.

### 3.3 Classification of breast cancer subtypes

One unique feature of PRIDE is that the D-Net constructed in a regression model can be easily extended to conduct classification of group labels, which cannot be achieved in previous DE- or IndE-based methods. In the Bayesian model, once the logistic regression model is trained, the probability of an individual being in group can be estimated based on the Bayesian prediction distribution.

This is demonstrated in a breast cancer study with data downloaded from the UCSC Xena TCGA Hub. The RNA sequencing expression profile was generated from IlluminaHiSeq platform with log2 transformed values of the RSEM-normalized count. Two subtypes Luminal-A (341 subjects) and Luminal-B (124 subjects) were selected for binary classification and prediction. The TP53 pathway with 57 nodes from the KEGG platform was adopted for the following analysis. The test set was based on a randomly selected 50 subjects, 25 from each group, and the remaining 415 were used to train the model. This procedure is repeated 100 times to calculate classification accuracy.

**Figure 6.**
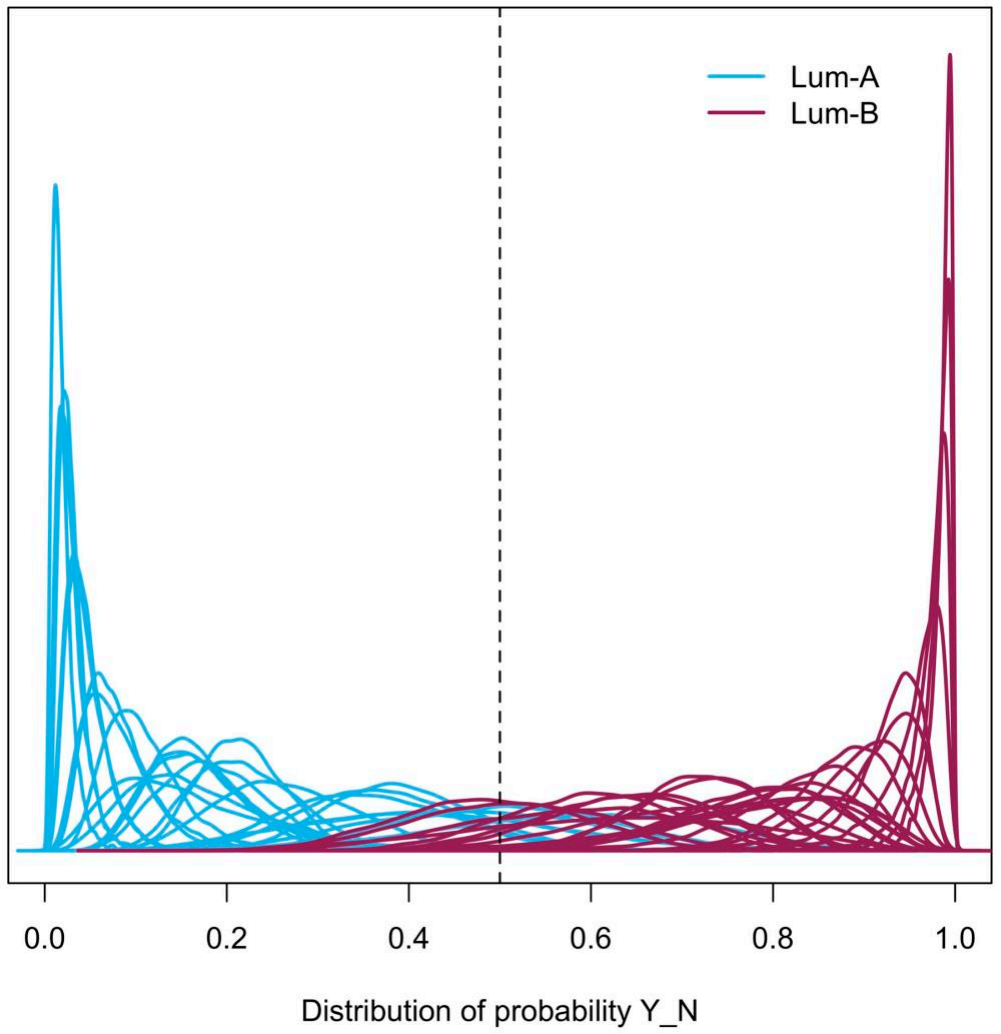
Posterior distribution of the classification probability. The curves are the posterior distributions of the classification probability for each subject in one of the replications.

The estimated posterior distributions of the label probability for 50 testing subjects in one of the replications are demonstrated in Fig. 6. Most distributions provided decisive evidence and correct identification of the group label. The classification accuracy of PRIDE was 0.86, with performance comparable to S-Lasso (0.87) and S-Ridge (0.87). The corresponding F1-scores were 0.86, 0.87, and 0.88 for the Bayesian model, S-Lasso, and S-Ridge, respectively. Though the respective performance of these logistic models was similar, PRIDE can provide more probabilistic information if needed. Detailed explanation of the Bayesian prediction distribution and estimates based on the MCMC method are in Supplementary notes T2.

## 4 Discussion

Although the differential network analysis has been an active research topic in recent decades, most algorithms focused on the test of the existence of differential edge. The uncertainty of the existence, however, cannot be quantified under such setting. In contrast, this uncertainty can be modelled intuitively with posterior probability under the Bayesian approach. In addition, among competing differential edges, their relative influence on the response variable can be investigated via the Bayesian probability, leading to the construction of a priority list. These goals can be achieved only when the estimation approach is implemented.

With the emphasis shifted from testing differential edges to estimation inference in genetic network analysis, this article adopts the perspective of the DE-based methods and proposes a Bayesian approach to estimate D-Net so that the edge existence and its association with the phenotype can be evaluated stochastically. This PRIDE approach is conducted in a regression model with detection of interactions, and therefore it can be easily extended to problems of classification and prediction with posterior probabilities. The simulations showed that, if only the deterministic decision of D-Net is of interest, PRIDE performs comparably or better than current methods. The advantages of PRIDE, however, are the other utilities it provides—for instance, the ability to detect differential intensity between two groups, such as JAK1-STAT3 demonstrated in the GBM study; to identify the sub-network in the D-Net; to prioritize the differential edges with probabilities; and to predict class labels. These advantages make PRIDE a complementary tool in differential network construction. When determining the final D-Net, the threshold 0.5 for the probability of existence is adopted. Other values can certainly be considered as alternatives. However, it should be noted that if the value is chosen larger than 0.5, then the sparsity would be even smaller than observed here, leading to lower F1-score. Replications from the simulation studies M1 and M2 were randomly taken to evaluate this influence for PRIDE and EB where probability measures are available to be compared with the threshold. The results show that 0.5 is a reasonable choice for PRIDE in both M1 and M2 but not for EB (Supplementary Fig. S5). Additionally, the network structures used in the simulation studies are taken from KEGG and PPI. Although the findings here indicate dependence on the structure, this conclusion is limited to the specific cases and structures investigated in this research.

We recognize that the PRIDE has some limitations. First, implementing the MCMC algorithm to generate the posterior samples limits the scalability of PRIDE to analyze large number of gene nodes. For instance, it takes nearly an hour to estimate 100 interactions when running the Bayesian computation in R on an ordinary desktop with intel core i7 processor (Supplementary Table S3). Therefore, the PRIDE may not be the priority method to use when screening a large number of interactions. Specifically, the PRIDE would be suitable for pathway analysis, in which the number of gene nodes ranges between 30 and 100. Alternatively, some recently proposed algorithms for fast discovery interactions in the Bayesian paradigm (Agrawal et al. 2019) or the software MultiBUGS (Goudie et al. 2020) with parallel implementation of MCMC could be implemented to increase the scalability of PRIDE. Second, we note that PRIDE did not achieve the lowest FDR among competing methods. It was larger than that of D-Trace, though smaller than all others. This is due to the lower sensitivity and smaller number of false positives of D-Trace. In practice, we suggest to examine the difference in D-Net obtained by D-Trace and PRIDE by evaluating the corresponding edge in each group-specific network like the current GBM study, and then using the posterior probability and expert information to determine if the differential edge should be included in the final D-Net. Additionally, independent filtering in the two-stage testing procedure (Dai et al. 2012) and multiple testing of the D-Net (Xia et al. 2015) may be incorporated into PRIDE to control the false discovery rate. Furthermore, we note that the PRIDE is not designed for directed graphs. The current setting cannot derive nor recover the original interconnectivity of nodes in each group-specific network. If this information is of interest, the indirect estimation-based approach may be favored over the current DE-based method for further extension.

It is worth noting that the PRIDE framework can be applied with other screening procedures. For instance, interaction screening (Fan et al. 2015), sparse and low-rank screening (Hung et al. 2016), and partial correlation screening (Wang and Chen 2020) have been proposed to efficiently target the interactions to be estimated under ultrahigh-dimensional scenarios. However, it would be expected to observe consistency to some degree and the resulting sets of candidate edges may contain many common elements. Systematic studies to investigate their differences would be worth pursuing. Furthermore, the idea of PRIDE may be applied in the study of differential causal effects (CE) recently proposed (Tian et al. 2016, Wang et al. 2018, Jablonski et al. 2022) to extend their approaches from testing CE to estimation of CE. In summary, the Bayesian approach to differential network analysis has opened up a promising research direction where the D-Net can be utilized in both association and classification studies.

The final remark relates to the term “gene-gene interaction” and “co-expression pattern,” which are often used interchangeably in bioinformatic and biological studies. The meaning, however, can vary across disciplines. For example, the DGCA defines the D-Net by the rewiring pattern of the co-expression pattern and by the difference in the correlation strength. In PRIDE, the D-Net is identified by the detection of gene-gene interaction, where the strength of the interaction is associated with the difference in conditional correlation. Although similarities between these two approaches have been observed in both simulation and real data analysis, one should be aware that the meaning of the D-Net constructed by the two methods is not identical. Future studies are warrant in formulating a more general framework to incorporate both perspectives.

## Supplementary Material

vbad172_Supplementary_Data

## Data Availability

All the data analyzed in this research can be downloaded at https://xenabrowser.net/datapages/. The R code for implementing PRIDE is available at https://github.com/YJGene0806/PRIDE_Code.
