# Supplementary material for: A Bayesian approach to differential edges with probabilistic interactions: applications in association and classification
Source: Bioinform Adv. 2023 Nov 24;3(1):vbad172. doi: 10.1093/bioadv/vbad172 (PMC10713123; doi:10.1093/bioadv/vbad172)
Supplement: vbad172_Supplementary_Data [file vbad172_supplementary_data.pdf]

Supplementary materials

Table S1. Detailed simulation settings of scenario M1. The last column is the value of the difference in partial correlation if it exists.

	P ($\binom{P}{2}$)	Gene nodes removed	Number of differential edges (sparsity)	Group1: Number of edges (sparsity)	Group2: Number of edges (sparsity)	Difference in partial correlation
M1.1: KEGG JAK-STAT	32 (496)	STAT1	17 (0.034)	34 (0.069)	17 (0.034)	0.219
M1.2: PPI EGFR	51 (1275)	EGFR	16 (0.013)	93 (0.073)	77 (0.060)	0.206
M1.3: KEGG MAPK	115 (6555)	MAPK14	15 (0.002)	167 (0.025)	152 (0.023)	0.196
M1.4: KEGG MAPK	115 (6555)	MAPK14+MAPK8	15+15=30 (0.005)	167 (0.025)	137 (0.021)	0.196

Table S2. Detailed simulation settings of M2. The intensity in the last two columns is the partial correlation between paired nodes, and the intensity of the remaining edge is the same as that in M1.

	P ($\binom{P}{2}$)	Nodes with different intensity	Number of differential edges (sparsity)	Group1: Number of edges (sparsity)	Group2: Number of edges (sparsity)	Group1: Intensity of edges	Group2: Intensity of edges
M2.1: KEGG JAK-STAT	32 (496)	STAT1	17 (0.034)	34 (0.069)	34 (0.069)	0.11	-0.11
M2.2: PPI EGFR	51 (1275)	EGFR	16 (0.013)	93 (0.073)	93 (0.073)	0.11	-0.11
M2.3: KEGG MAPK	115 (6555)	MAPK14	15 (0.002)	167 (0.025)	167 (0.025)	0.11	-0.11
M2.4: KEGG MAPK	115 (6555)	MAPK14+MAPK8	15+15=30 (0.005)	167 (0.025)	167 (0.025)	0.11	-0.11

Table S3. The number of interactions passing the screening procedure before the implementation of PRIDE under scenarios M1 and M2. The computation time of PRIDE to estimate the differential network for one simulated dataset is shown in the last column.

	$P(\binom{P}{2})$	Number of differential edges (network sparsity)	Number of screening edges (screening proportion)	Computation time
JAK-STAT	32 (496)	17 (3.4%)	25 (5%)	14 mins
EGFR	51 (1275)	16 (1.3%)	32 (2.5%)	19 mins
MAPK	115 (6555)	15 (0.2%)	33 (0.5%)	19 mins
MAPK	115 (6555)	30 (0.5%)	66 (1%)	50 mins

Note: The number of candidate interactions is about two times the number of true differential edges. The computation time of PRIDE is based on 5,000 generated posterior samples (with 5,000 burn-ins, 10,000 iterations and 20 thining).

Criteria used below to measure the performance of simulation results

To measure and compare the performance of PRIDE and other methods, we consider the number of true positives (TP), number of false positives (FP), sensitivity (SEN), specificity (SPE), false discovery proportion (FDP), Matthew correlation coefficient (MCC), and the F1-score (F1), defined as follows:

$$\mathrm{SEN} = \frac{\mathrm{TP}}{\mathrm{TP}+\mathrm{FN}}, \quad \mathrm{SPE} = \frac{\mathrm{TN}}{\mathrm{TN}+\mathrm{FP}}, \quad \mathrm{FDP} = \frac{\mathrm{FP}}{\mathrm{TP}+\mathrm{FP}},$$

$$\mathrm{MCC} = \frac{\mathrm{TP}\times\mathrm{TN}-\mathrm{FP}\times\mathrm{FN}}{\sqrt{(\mathrm{TP}+\mathrm{FP})(\mathrm{TP}+\mathrm{FN})(\mathrm{TN}+\mathrm{FP})(\mathrm{TN}+\mathrm{FN})}},$$

$$\mathrm{F1} = \frac{2\mathrm{TP}}{2\mathrm{TP}+\mathrm{FP}+\mathrm{FN}},$$

A larger value of MCC and F1 indicates better performance.

Table S4. Summarized simulation performance of M1.1–M1.4. Values are the mean (SE) based on 100 replications. The false discovery rate (FDR) is defined as the average value of the false discovery proportion (FDP), where FDP is defined as the ratio of FP to the sum of TP and FP. The number of true differential edges is 17 in M1.1, 16 in M1.2, 15 in M1.3, and 30 in M1.4.

**M1.1**	TP	FP	SEN	SPE	FDR	MCC	F1
PRIDE	13.27 (1.75)	8.82 (1.53)	0.78 (0.10)	0.98 (<0.01)	0.40 (0.07)	0.67 (0.08)	0.68 (0.08)
LASSO	11.94 (1.96)	37.4 (16.79)	0.70 (0.12)	0.92 (0.04)	0.73 (0.10)	0.40 (0.09)	0.38 (0.09)
S-LASSO	14.31 (1.56)	9.66 (1.25)	0.84 (0.09)	0.98 (<0.01)	0.40 (0.05)	0.70 (0.07)	0.69 (0.07)
D-Trace	12.61 (1.90)	5.13 (2.29)	0.74 (0.11)	0.99 (<0.01)	0.28 (0.10)	0.72 (0.09)	0.73 (0.08)
GLasso	16.94 (0.24)	34.01 (6.79)	>0.99 (0.01)	0.93 (0.01)	0.66 (0.04)	0.56 (0.04)	0.50 (0.05)
M&B	16.93 (0.26)	13.09 (3.79)	>0.99 (0.02)	0.97 (0.01)	0.43 (0.07)	0.74 (0.05)	0.72 (0.06)
SPACE	16.85 (0.36)	15.49 (3.69)	0.99 (0.02)	0.97 (0.01)	0.47 (0.06)	0.71 (0.04)	0.69 (0.05)
BH	16.48 (0.78)	31.96 (12.36)	0.97 (0.05)	0.93 (0.03)	0.64 (0.09)	0.57 (0.07)	0.52 (0.09)
Bonferroni	14.46 (1.53)	4.30 (2.69)	0.85 (0.09)	0.99 (0.01)	0.21 (0.11)	0.81(0.07)	0.81 (0.07)
EB	15.92 (1.10)	10.74 (8.83)	0.94 (0.06)	0.98 (0.02)	0.34 (0.20)	0.77 (0.13)	0.76 (0.14)
**M1.2**							
PRIDE	11.02 (1.70)	13.45 (2.06)	0.69 (0.11)	0.99 (<0.01)	0.55 (0.06)	0.55 (0.08)	0.54 (0.07)
LASSO	8.76 (1.58)	39.30 (22.05)	0.55 (0.10)	0.97 (0.02)	0.78 (0.10)	0.32 (0.09)	0.30 (0.10)
S-LASSO	11.00 (1.67)	15.34 (2.15)	0.69 (0.10)	0.99 (<0.01)	0.58 (0.06)	0.53 (0.08)	0.52 (0.07)
D-Trace	10.28 (1.70)	8.46 (3.58)	0.64 (0.11)	0.99 (<0.01)	0.44 (0.11)	0.59 (0.09)	0.59 (0.09)
GLasso	15.18 (0.69)	120.01 (17.86)	0.95 (0.04)	0.90 (0.01)	0.89 (0.01)	0.31 (0.03)	0.20 (0.02)
M&B	14.68 (0.51)	45.22 (5.94)	0.92 (0.03)	0.96 (<0.01)	0.75 (0.02)	0.47 (0.03)	0.39 (0.03)
SPACE	14.83 (0.40)	51.87 (6.09)	0.93 (0.03)	0.96 (<0.01)	0.78 (0.02)	0.44 (0.02)	0.36 (0.03)
BH	14.88 (1.06)	85.30 (37.60)	0.93 (0.07)	0.93 (0.03)	0.83 (0.06)	0.38 (0.07)	0.28 (0.09)
Bonferroni	12.25 (1.65)	13.50 (5.87)	0.77 (0.10)	0.99 (<0.01)	0.50 (0.11)	0.61 (0.08)	0.59 (0.08)
EB	14.22 (1.05)	37.62 (14.51)	0.89 (0.07)	0.97 (0.01)	0.71 (0.08)	0.50 (0.07)	0.44 (0.09)
**M1.3**							
PRIDE	10.86 (1.78)	14.10 (1.95)	0.72 (0.12)	>0.99 (<0.01)	0.56 (0.06)	0.56 (0.08)	0.54 (0.08)
LASSO	7.87 (1.66)	44.22 (31.70)	0.52 (0.11)	0.99 (<0.01)	0.79 (0.12)	0.31 (0.10)	0.28 (0.11)
S-LASSO	11.28 (1.76)	15.60 (2.11)	0.75 (0.12)	>0.99 (<0.01)	0.58 (0.06)	0.56 (0.08)	0.54 (0.08)
D-Trace	8.22 (1.66)	5.27 (2.57)	0.55 (0.11)	>0.99 (<0.01)	0.37 (0.13)	0.58 (0.10)	0.58 (0.10)
GLasso	14.98 (0.14)	140.29 (20.30)	>0.99 (0.01)	0.98 (<0.01)	0.90 (0.01)	0.31 (0.02)	0.18 (0.02)
M&B	14.97 (0.17)	69.92 (8.96)	>0.99 (0.01)	0.99 (<0.01)	0.82 (0.02)	0.42 (0.02)	0.30 (0.03)
SPACE	14.98 (0.14)	87.00 (9.55)	>0.99 (0.01)	0.99 (<0.01)	0.85 (0.01)	0.38 (0.02)	0.26 (0.02)
BH	14.18 (0.86)	47.09 (21.55)	0.95 (0.06)	0.99 (<0.01)	0.74 (0.09)	0.49 (0.08)	0.40 (0.10)
Bonferroni	11.64 (1.53)	10.05 (4.92)	0.78 (0.10)	0.99 (<0.01)	0.44 (0.12)	0.65 (0.08)	0.64 (0.08)

EB	14.40 (0.71)	40.14 (14.58)	0.96 (0.05)	0.99 (<0.01)	0.72 (0.07)	0.51 (0.07)	0.43 (0.09)
**M1.4**							
PRIDE	20.25 (2.14)	31.43 (3.35)	0.68 (0.07)	>0.99 (<0.01)	0.61 (0.04)	0.51 (0.05)	0.50 (0.05)
LASSO	13.78 (2.56)	64.07 (32.83)	0.46 (0.09)	0.99 (0.01)	0.79 (0.10)	0.30 (0.07)	0.27 (0.08)
S-LASSO	19.49 (2.07)	28.81 (3.58)	0.65 (0.07)	>0.99 (<0.01)	0.60 (0.04)	0.51 (0.05)	0.50 (0.05)
D-Trace	18.92 (2.26)	19.56 (5.48)	0.63 (0.08)	>0.99 (<0.01)	0.50 (0.08)	0.56 (0.06)	0.55 (0.06)
GLasso	29.32 (0.84)	139.07 (16.63)	0.98 (0.03)	0.98 (<0.01)	0.82 (0.02)	0.41 (0.02)	0.30 (0.02)
M&B	28.89 (1.08)	68.03 (8.26)	0.96 (0.04)	0.99 (<0.01)	0.70 (0.02)	0.53 (0.03)	0.46 (0.03)
SPACE	29.24 (0.93)	87.27 (7.31)	0.97 (0.03)	0.99 (<0.01)	0.75 (0.02)	0.49 (0.02)	0.40 (0.02)
BH	25.68 (2.02)	77.39 (28.97)	0.86 (0.07)	0.99 (<0.01)	0.73 (0.08)	0.47 (0.06)	0.40 (0.08)
Bonferroni	18.77 (2.25)	15.72 (6.55)	0.63 (0.07)	0.99 (<0.01)	0.44 (0.10)	0.59 (0.06)	0.59 (0.06)
EB	26.24 (1.74)	53.63 (18.46)	0.87 (0.06)	0.99 (<0.01)	0.65 (0.08)	0.54 (0.06)	0.49 (0.08)

Table S5. Summarized simulation results for scenarios M2.1–M2.4. Values are the mean (SE) based on 100 replications. False discovery rate (FDR) is defined as the average value of FDP. The number of true differential edges is 17 in M2.1, 16 in M2.2, 15 in M2.3, and 30 in M2.4.

**M2.1**	TP	FP	SEN	SPE	FDR	MCC	F1
PRIDE	11.03 (1.58)	13.05 (1.68)	0.65 (0.09)	0.97 (<0.01)	0.54 (0.07)	0.53 (0.08)	0.54 (0.08)
LASSO	10.45 (2.45)	25.01 (16.19)	0.61 (0.14)	0.95 (0.03)	0.64 (0.17)	0.43 (0.09)	0.42 (0.10)
S-LASSO	11.34 (1.60)	13.56 (1.62)	0.67 (0.09)	0.97 (<0.01)	0.54 (0.06)	0.53 (0.08)	0.54 (0.08)
D-Trace	11.26 (1.78)	12.27 (3.70)	0.66 (0.10)	0.97 (0.01)	0.51 (0.08)	0.55 (0.08)	0.56 (0.08)
GLasso	4.58 (1.60)	9.81 (3.22)	0.27 (0.09)	0.98 (0.01)	0.67 (0.12)	0.27 (0.10)	0.29 (0.09)
M&B	4.50 (1.57)	9.07 (3.22)	0.26 (0.09)	0.98 (0.01)	0.66 (0.12)	0.28 (0.10)	0.29 (0.10)
SPACE	6.17 (1.85)	13.01 (3.79)	0.36 (0.11)	0.97 (0.01)	0.67 (0.10)	0.32 (0.10)	0.34 (0.10)
BH	3.67 (2.19)	0.30 (0.59)	0.22 (0.13)	>0.99 (<0.01)	NA	NA	0.33 (0.17)
Bonferroni	2.04 (1.23)	0.04 (0.20)	0.12 (0.07)	>0.99 (<0.01)	NA	NA	0.21 (0.11)
EB	0.62 (0.80)	0.03 (0.17)	0.04 (0.05)	>0.99 (<0.01)	NA	NA	0.07 (0.08)
**M2.2**							
PRIDE	9.73 (1.90)	18.07 (2.50)	0.61 (0.12)	0.99 (<0.01)	0.65 (0.07)	0.45 (0.09)	0.44 (0.08)
LASSO	7.90 (2.43)	26.34 (20.76)	0.49 (0.15)	0.98 (0.02)	0.67 (0.20)	0.36 (0.10)	0.34 (0.10)
S-LASSO	9.89 (1.83)	19.83 (2.47)	0.62 (0.11)	0.98 (<0.01)	0.67 (0.06)	0.44 (0.08)	0.43 (0.08)
D-Trace	7.86 (1.96)	6.87 (2.61)	0.49 (0.12)	0.99 (<0.01)	0.46 (0.12)	0.51 (0.10)	0.51 (0.10)
GLasso	3.67 (1.58)	52.05 (7.76)	0.23 (0.10)	0.96 (0.01)	0.93 (0.03)	0.10 (0.05)	0.10 (0.04)
M&B	3.31 (1.45)	34.90 (4.92)	0.21 (0.09)	0.97 (<0.01)	0.91 (0.04)	0.12 (0.06)	0.12 (0.05)
SPACE	4.39 (1.80)	41.13 (5.84)	0.27 (0.11)	0.97 (<0.01)	0.90 (0.04)	0.15 (0.07)	0.14 (0.06)
BH	4.42 (2.08)	1.77 (1.95)	0.28 (0.13)	>0.99 (<0.01)	0.23 (0.20)	0.44 (0.12)	0.38 (0.14)

Bonferroni	2.86 (1.21)	0.60 (0.92)	0.18 (0.08)	>0.99 (<0.01)	0.14 (0.19)	0.38 (0.10)	0.29 (0.10)
EB	2.51 (1.37)	0.72 (1.16)	0.16 (0.09)	>0.99 (<0.01)	NA	NA	0.25 (0.12)
**M2.3**							
PRIDE	9.68 (1.41)	20.59 (1.97)	0.65 (0.09)	>0.99 (<0.01)	0.68 (0.05)	0.45 (0.07)	0.43 (0.06)
LASSO	7.49 (1.87)	38.13 (30.55)	0.50 (0.12)	0.99 (<0.01)	0.74 (0.18)	0.33 (0.10)	0.30 (0.11)
S-LASSO	9.86 (1.40)	21.17 (1.77)	0.66 (0.09)	>0.99 (<0.01)	0.68 (0.04)	0.46 (0.06)	0.43 (0.06)
D-Trace	7.35 (1.55)	6.10 (1.55)	0.49 (0.10)	>0.99 (<0.01)	0.44 (0.13)	0.52 (0.10)	0.52 (0.10)
GLasso	3.29 (1.37)	82.43 (9.08)	0.22 (0.09)	0.99 (<0.01)	0.96 (0.02)	0.09 (0.04)	0.07 (0.03)
M&B	3.16 (1.32)	62.66 (6.88)	0.21 (0.09)	0.99 (<0.01)	0.95 (0.02)	0.10 (0.04)	0.08 (0.03)
SPACE	4.62 (1.70)	78.75 (7.51)	0.31 (0.11)	0.99 (<0.01)	0.94 (0.02)	0.13 (0.05)	0.09 (0.03)
BH	5.15 (1.83)	1.63 (1.46)	0.34 (0.12)	>0.99 (<0.01)	NA	NA	0.46 (0.12)
Bonferroni	3.51 (1.44)	0.71 (0.77)	0.23 (0.10)	>0.99 (<0.01)	NA	NA	0.36 (0.12)
EB	1.86 (1.41)	0.50 (0.70)	0.12 (0.09)	>0.99 (<0.01)	NA	NA	0.20 (0.14)
**M2.4**							
PRIDE	16.93 (2.35)	45.78 (2.83)	0.56 (0.08)	0.99 (<0.01)	0.73 (0.04)	0.39 (0.05)	0.37 (0.05)
LASSO	13.03 (3.29)	51.68 (34.56)	0.43 (0.11)	0.99 (0.01)	0.74 (0.14)	0.31 (0.06)	0.29 (0.08)
S-LASSO	16.76 (2.26)	45.58 (3.33)	0.56 (0.08)	0.99 (<0.01)	0.73 (0.04)	0.38 (0.05)	0.36 (0.05)
D-Trace	10.35 (2.62)	5.89 (2.37)	0.34 (0.09)	>0.99 (<0.01)	0.36 (0.10)	0.47 (0.08)	0.44 (0.09)
GLasso	3.95 (1.87)	69.81 (9.06)	0.13 (0.06)	0.99 (<0.01)	0.95 (0.03)	0.08 (0.04)	0.08 (0.04)
M&B	3.96 (1.75)	57.06 (7.32)	0.13 (0.06)	0.99 (<0.01)	0.93 (0.03)	0.09 (0.04)	0.09 (0.04)
SPACE	7.01 (1.87)	73.88 (8.88)	0.23 (0.06)	0.99 (<0.01)	0.91 (0.02)	0.14 (0.04)	0.13 (0.03)
BH	5.42 (2.86)	0.97 (1.09)	0.18 (0.10)	>0.99 (<0.01)	NA	NA	0.29 (0.13)
Bonferroni	3.00 (1.81)	0.20 (0.47)	0.10 (0.06)	>0.99 (<0.01)	NA	NA	0.18 (0.10)
EB	1.48 (1.37)	0.20 (0.49)	0.05 (0.05)	>0.99 (<0.01)	NA	NA	0.09 (0.08)

Note: the “NA” value from differential correlation-based methods is due to the zero detection of true positive differential edge in some of the replications.

Table S6. Detailed settings of simulation scenario M3.

	P ($\binom{P}{2}$)	Number of differential edges (sparsity)	AR(2): Number of edges (sparsity)	AR(1): Number of edges (sparsity)
M3.1	25 (300)	23 (0.077)	47 (0.157)	24 (0.080)
M3.2	50 (1225)	48 (0.039)	97 (0.079)	49 (0.040)
M3.3	75 (2775)	73 (0.026)	147 (0.053)	174 (0.027)

Figure S1.

Results of additional simulations. The Y-axis denotes the average F1-score across 100 replications with error bar corresponding to the standard error. The group in blue represents the DE-based methods (PRIDE in light blue), pink indicates an SE-based methods, and yellow shows the results of correlation-based approaches. The number of differential edges equals 23 (*P*=25), 48 (*P*=50), and 73 (*P*=75).

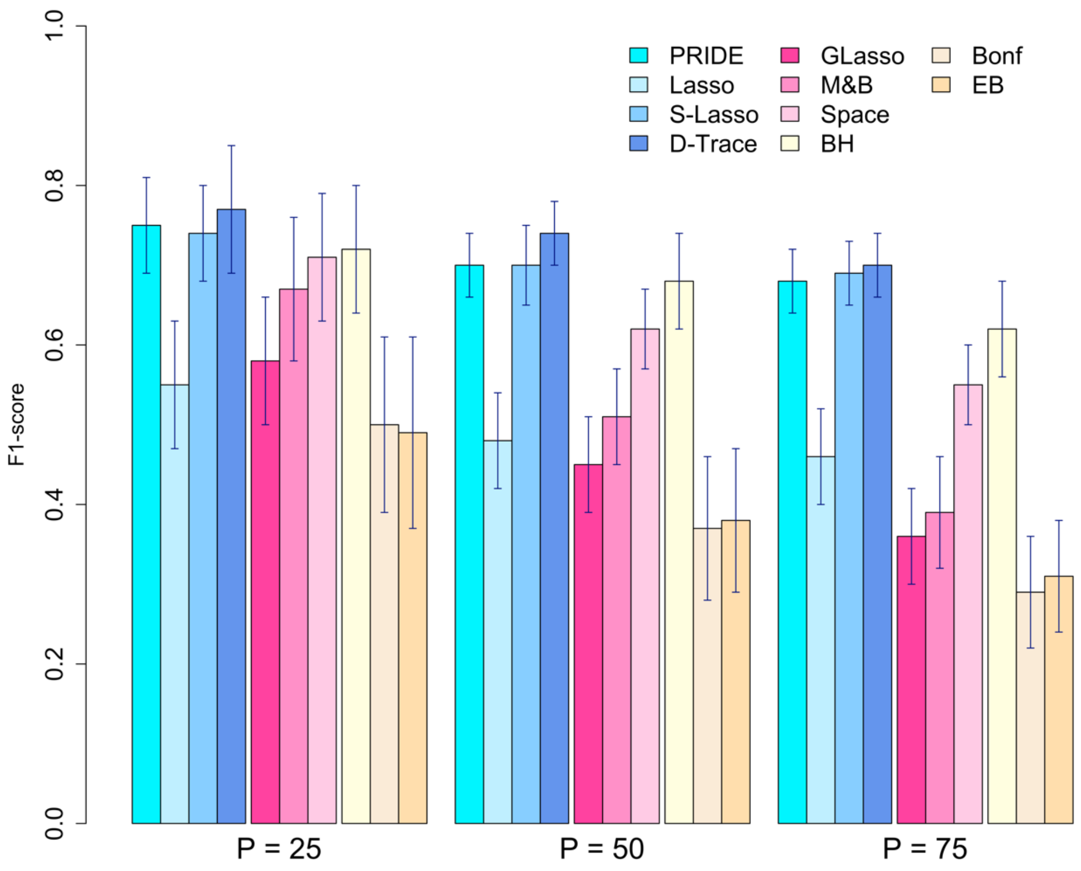

Table S7. Summarized results for simulations M3.1–M3.3. Values are the mean (SE) based on 100 replications. False discovery rate (FDR) is defined as the average value of the FDP. The numbers of true differential edges are 23, 47, and 73 for $P=25$, 50, and 75, respectively.

**M3.1**	TP	FP	SEN	SPE	FDR	MCC	F1
PRIDE	18.61 (1.64)	7.79 (1.98)	0.81 (0.07)	0.97 (0.01)	0.29 (0.07)	0.73 (0.07)	0.75 (0.06)
LASSO	20.73 (1.43)	33.25(11.52)	0.90 (0.06)	0.88 (0.04)	0.60 (0.09)	0.55 (0.08)	0.55 (0.08)

S-LASSO	19.29 (1.55)	9.52 (1.88)	0.84 (0.07)	0.97 (0.01)	0.33 (0.06)	0.73 (0.07)	0.74 (0.06)
D-Trace	15.96 (2.34)	2.34 (1.56)	0.69 (0.10)	0.99 (0.01)	0.12 (0.08)	0.76 (0.08)	0.77 (0.08)
GLasso	14.02 (2.86)	11.29 (2.99)	0.61 (0.12)	0.96 (0.01)	0.44 (0.07)	0.54 (0.09)	0.58 (0.08)
M&B	15.66 (2.86)	7.82 (2.69)	0.68 (0.12)	0.97 (0.01)	0.33 (0.09)	0.65 (0.10)	0.67 (0.09)
SPACE	16.95 (2.42)	7.80 (2.75)	0.74 (0.11)	0.97 (0.01)	0.31 (0.08)	0.69 (0.09)	0.71 (0.08)
BH	15.48 (2.94)	4.11 (2.29)	0.67 (0.13)	0.99 (0.01)	0.20 (0.08)	0.71 (0.08)	0.72 (0.08)
Bonferroni	8.09 (2.42)	0.55 (0.73)	0.35 (0.11)	>0.99 (<0.01)	0.06 (0.08)	0.55 (0.09)	0.50 (0.11)
EB	7.74 (2.46)	0.54 (0.67)	0.34 (0.11)	>0.99 (<0.01)	0.06 (0.08)	0.54 (0.10)	0.49 (0.12)
**M3.2**							
PRIDE	36.43 (2.43)	20.41 (2.83)	0.76 (0.05)	0.98 (<0.01)	0.36 (0.04)	0.68 (0.05)	0.70 (0.04)
LASSO	38.81 (2.56)	77.19 (21.74)	0.81 (0.05)	0.93 (0.02)	0.65 (0.07)	0.50 (0.05)	0.48 (0.06)
S-LASSO	37.02 (2.42)	20.52 (2.87)	0.77 (0.05)	0.98 (<0.01)	0.36 (0.04)	0.69 (0.05)	0.70 (0.05)
D-Trace	33.00 (2.52)	8.69 (2.76)	0.69 (0.05)	0.99 (<0.01)	0.21 (0.05)	0.73 (0.04)	0.74 (0.04)
GLasso	21.48 (3.83)	24.72 (3.45)	0.45 (0.08)	0.98 (<0.01)	0.54 (0.05)	0.43 (0.06)	0.45 (0.06)
M&B	23.31 (3.50)	19.44 (4.19)	0.49 (0.07)	0.98 (<0.01)	0.45 (0.07)	0.50 (0.06)	0.51 (0.06)
SPACE	29.67 (3.11)	17.63 (3.47)	0.62 (0.06)	0.99 (<0.01)	0.37 (0.05)	0.61 (0.05)	0.62 (0.05)
BH	27.73 (3.92)	5.96 (2.60)	0.58 (0.08)	0.99 (<0.01)	0.17 (0.06)	0.68 (0.05)	0.68 (0.06)
Bonferroni	11.34 (3.21)	0.53 (0.66)	0.24 (0.07)	>0.99 (<0.01)	0.04 (0.05)	0.46 (0.07)	0.37 (0.09)
EB	11.61 (3.17)	1.01 (1.08)	0.24 (0.07)	>0.99 (<0.01)	0.08 (0.09)	0.46 (0.07)	0.38 (0.09)
**M3.3**							
PRIDE	53.13 (3.56)	29.15 (3.21)	0.73 (0.05)	0.99 (<0.01)	0.35 (0.04)	0.68 (0.04)	0.68 (0.04)
LASSO	52.91 (3.65)	106.11 (27.42)	0.72 (0.05)	0.96 (0.01)	0.66 (0.06)	0.48 (0.05)	0.46 (0.06)
S-LASSO	51.24 (3.45)	24.98 (3.65)	0.70 (0.05)	0.99 (<0.01)	0.33 (0.05)	0.68 (0.05)	0.69 (0.04)
D-Trace	49.45 (3.97)	18.16 (4.84)	0.68 (0.05)	0.99 (<0.01)	0.27 (0.05)	0.70 (0.04)	0.70 (0.04)
GLasso	25.35 (5.20)	40.76 (5.21)	0.35 (0.07)	0.98 (<0.01)	0.62 (0.06)	0.35 (0.06)	0.36 (0.06)
M&B	26.57 (5.49)	34.98 (5.16)	0.36 (0.08)	0.99 (<0.01)	0.57 (0.07)	0.38 (0.07)	0.39 (0.07)
SPACE	39.21 (4.56)	29.76 (5.95)	0.54 (0.06)	0.99 (<0.01)	0.43 (0.06)	0.54 (0.05)	0.55 (0.05)
BH	36.31 (5.96)	6.75 (3.47)	0.50 (0.08)	>0.99 (<0.01)	0.15 (0.06)	0.64 (0.05)	0.62 (0.06)
Bonferroni	12.65 (3.73)	0.38 (0.71)	0.17 (0.05)	>0.99 (<0.01)	0.03 (0.05)	0.40 (0.06)	0.29 (0.07)
EB	13.97 (3.79)	1.20 (1.10)	0.19 (0.05)	>0.99 (<0.01)	0.08 (0.06)	0.41 (0.06)	0.31 (0.07)

Figure S2. Venn diagram of the D-Net identified by various DE-based tools.

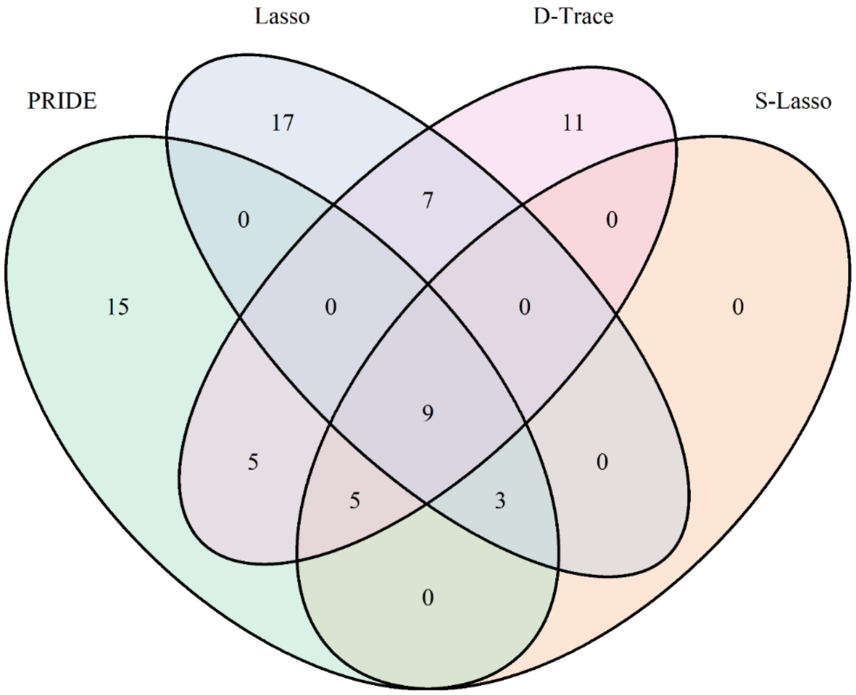

Figure S3. Venn diagram of the D-Net identified by PRIDE and various IndE-based tools.

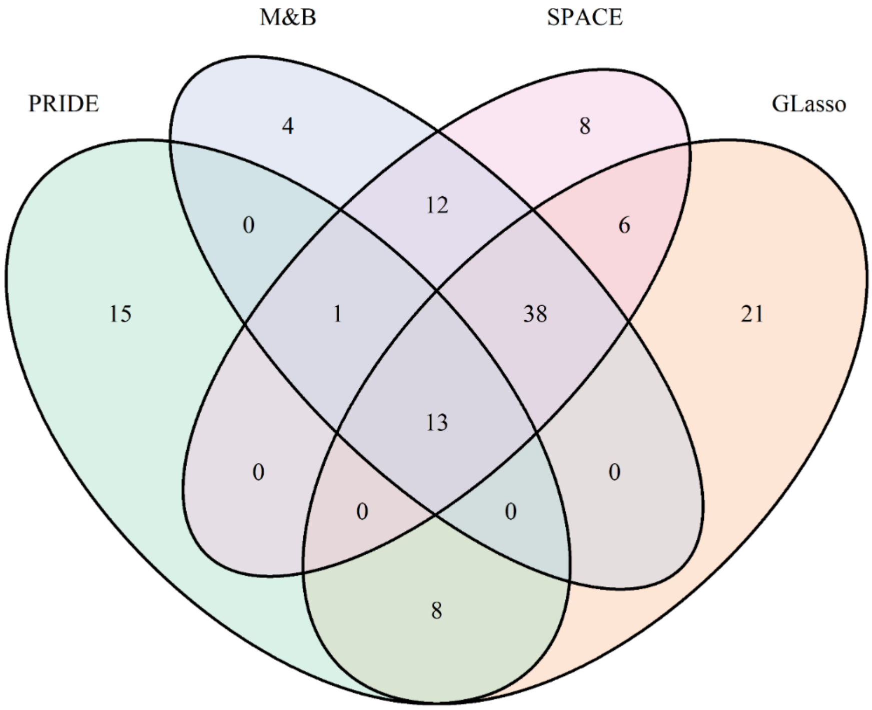

Figure S4. Venn diagram of the D-Net identified by PRIDE and various differential correlation-based tools.

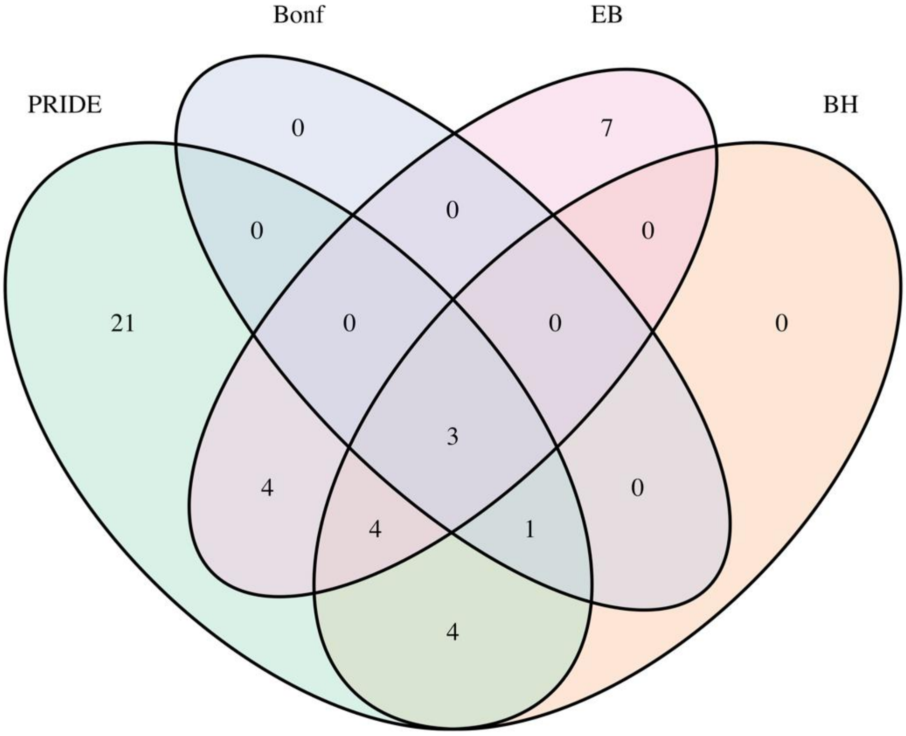

Figure S5. F1-score of PRIDE and EBcoexpress (EB) at different threshold values.

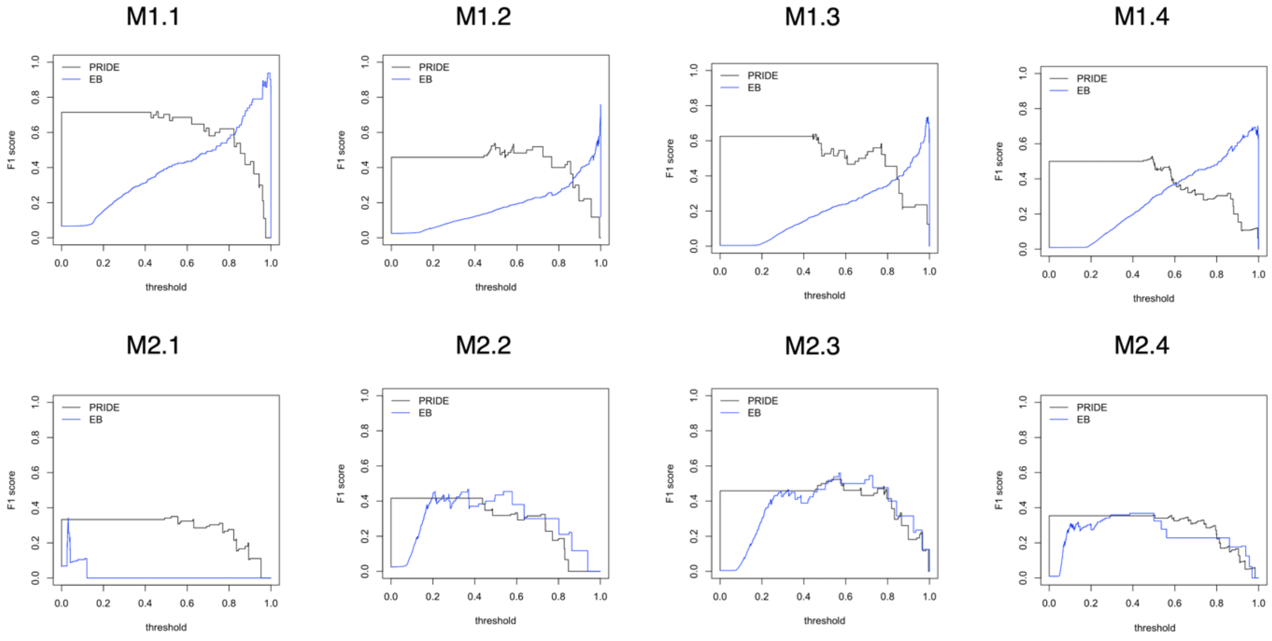

Supplementary notes T1: references that supported the biological insight of our findings in glioblastoma data analysis

Gheidari,F. et al. (2021). miR-424 induces apoptosis in glioblastoma cells and targets AKT1 and RAF1 oncogenes from the ERBB signaling pathway. European Journal of Pharmacology, 906, 174273.

Kim,J.E. et al. (2014). Stat3 activation in glioblastoma: Biochemical and therapeutic implications. Cancers, 6, Article 1.

Liu,Z. et al. (2014). MiR-7-5p is frequently downregulated in glioblastoma microvasculature and inhibits vascular endothelial cell proliferation by targeting RAF1. Tumor Biology, 35, 10177–10184.

Ou,A. et al. (2021). The role and therapeutic targeting of JAK/STAT signaling in glioblastoma. cancers, 13, Article 3.

Park,A.K. et al. (2019). Subtype-specific signaling pathways and genomic aberrations associated with prognosis of glioblastoma. Neuro-Oncology, 21, 59–70.

Qureshy,Z. et al. (2020). Targeting the JAK/STAT pathway in solid tumors. Journal of Cancer Metastasis and Treatment, 2020.

Sidaway,P. (2017). Glioblastoma subtypes revisited. Nature Reviews Clinical Oncology, 14, Article 10.

Steponaitis,G. and Tamasauskas,A. (2021). Mesenchymal and Proneural subtypes of

glioblastoma disclose branching based on gsc associated signature. International Journal of Molecular Sciences, 22, 4964.

Teo,W.-Y. et al. (2019). Relevance of a TCGA-derived glioblastoma subtype gene-classifier among patient populations. Scientific Reports, 9, Article 1.

The Cancer Genome Atlas Research Network. (2008). Comprehensive genomic characterization defines human glioblastoma genes and core pathways. Nature, 455(7216), 1061–1068.

Yang,H. et al. (2019). A thirteen-gene set efficiently predicts the prognosis of glioblastoma. Molecular Medicine Reports, 19, 1613–1621.

Supplementary notes T2: computational details for the classification of the TCGA breast cancer dataset

The predictive distribution is defined as $f(Y_N \mid Y_O, X_O, X_N)$, which can be written as

$$f(Y_N \mid Y_O, X_O, X_N) = \int_{\Theta} f(Y_N, \Theta \mid Y_O, X_O, X_N) d\Theta = \int_{\Theta} f(Y_N \mid \Theta, Y_O, X_O, X_N) \times \pi(\Theta \mid Y_O, X_O, X_N) d\Theta$$

where $\Theta = \{\boldsymbol{\beta}, \boldsymbol{M}\}$ contains all parameters. Specifically, $\boldsymbol{\beta}$ represents the interaction coefficients and $\boldsymbol{M}$ denotes the model generated in each MCMC sampling. In our case, the model M denotes the generating D-Net structure in each iteration. The above equation can be written as

$$f(Y_N \mid Y_O, X_O, X_N) = \int_\Theta f(Y_N \mid \Theta, X_N) \times \pi(\Theta \mid Y_O, X_O) d\Theta$$
$$= \int_\Theta f(Y_N \mid \beta, M, X_N) \times \pi(\beta \mid M, Y_O, X_O) \times \pi(M \mid Y_O, X_O) d\Theta \, .$$

The integration on the right-hand side can be computed by Monte Carlo integration. Based on the generated S posterior samples for each β_{jk} and γ_{jk}, $1 \le j < k \le P$, the unnormalized model probability is calculated as

$P^*(M_i \mid .) = \prod_{(j,k)} \left[I(\gamma_{jk}^i = 1) \times P(\gamma_{jk} = 1 \mid .) + I(\gamma_{jk}^i = 0) \times \left\{ 1 - P(\gamma_{jk} = 1 \mid .) \right\} \right]$, where $P^*(M_i \mid .)$ represents the unnormalized model probability for the ith generating posterior samples, $i = 1, 2, ..., S$. The posterior probability of differential edges $P(\gamma_{jk} = 1 \mid .)$ is calculated as $P(\gamma_{jk} = 1 \mid .) = \frac{\sum_{i=1}^{S} I(\gamma_{jk} = 1 \mid .)}{S}$. The final normalized model probability for each generating posterior sample is defined as $P(M_i \mid .) = \frac{P^*(M_i \mid .)}{\sum_{i=1}^{S} P^*(M_i \mid .)}$ to ensure the sum of all posterior model probabilities equals 1. Finally, the posterior probability $P(Y_N = 1 \mid .)$ is computed:

$P(Y_N = 1 \mid .) = \sum_{i=1}^{S} P(Y_N^i = 1 \mid ., M_i) \times P(M_i \mid .)$, where $P(Y_N^i = 1 \mid .) = \frac{\exp(\hat{\boldsymbol{\beta}}^{i^T} \boldsymbol{X}_N)}{1 + \exp(\hat{\boldsymbol{\beta}}^{i^T} \boldsymbol{X}_N)}$

and $\hat{\boldsymbol{\beta}}^i = \left\{ \beta_{jk}^i \mid 1 \le j < k \le p \right\}$, $i = 1, 2, ..., S$.
